# Lithium-Responsive Seizure-Like Hyperexcitability Is Caused by a Mutation in the *Drosophila* Voltage-Gated Sodium Channel Gene *paralytic*

**DOI:** 10.1523/ENEURO.0221-16.2016

**Published:** 2016-11-10

**Authors:** Garrett A. Kaas, Junko Kasuya, Patrick Lansdon, Atsushi Ueda, Atulya Iyengar, Chun-Fang Wu, Toshihiro Kitamoto

**Affiliations:** 1 Interdisciplinary Graduate Program in Genetics, University of Iowa, IA 52242, USA; 2 Department of Anesthesia, Carver College of Medicine, University of Iowa, IA 52242; 3 Department of Biology, College of Liberal Arts and Sciences, University of Iowa, IA 52242; 4 Interdisciplinary Graduate Program in Neuroscience, University of Iowa, IA 52242

**Keywords:** Drosophila, lithium, neurogenetics, seizure, voltage-gated sodium channel

## Abstract

*Shudderer* (*Shu*) is an X-linked dominant mutation in *Drosophila melanogaster* identified more than 40 years ago. A previous study showed that *Shu* caused spontaneous tremors and defects in reactive climbing behavior, and that these phenotypes were significantly suppressed when mutants were fed food containing lithium, a mood stabilizer used in the treatment of bipolar disorder (Williamson, 1982). This unique observation suggested that the *Shu* mutation affects genes involved in lithium-responsive neurobiological processes. In the present study, we identified *Shu* as a novel mutant allele of the voltage-gated sodium (Na_v_) channel gene *paralytic* (*para*). Given that hypomorphic *para* alleles and RNA interference–mediated *para* knockdown reduced the severity of *Shu* phenotypes, *Shu* was classified as a *para* hypermorphic allele. We also demonstrated that lithium could improve the behavioral abnormalities displayed by other Na_v_ mutants, including a fly model of the human generalized epilepsy with febrile seizures plus. Our electrophysiological analysis of *Shu* showed that lithium treatment did not acutely suppress Na_v_ channel activity, indicating that the rescue effect of lithium resulted from chronic physiological adjustments to this drug. Microarray analysis revealed that lithium significantly alters the expression of various genes in *Shu*, including those involved in innate immune responses, amino acid metabolism, and oxidation-reduction processes, raising the interesting possibility that lithium-induced modulation of these biological pathways may contribute to such adjustments. Overall, our findings demonstrate that Na_v_ channel mutants in *Drosophila* are valuable genetic tools for elucidating the effects of lithium on the nervous system in the context of neurophysiology and behavior.

## Significance Statement

The alkaline metal lithium has been used as one of the most effective mood-stabilizing agents for bipolar disorder for more than 60 years. Although a number of molecular targets for lithium have been proposed, the neural mechanisms underlying lithium action still remain unclear. Here we show that lithium treatment significantly reduced the severity of seizure-like hyperexcitability displayed by hypermorphic alleles of the *Drosophila* Na_v_ channel gene. Our studies indicate that suppression of mutant phenotypes is achieved through lithium-induced physiological adjustments, leading to compensation of defects caused by mutated Na_v_ channels. These findings provide novel insight into the effects of the mood-stabilizing agent lithium on neural function and behavior and may ultimately contribute to a clearer understanding of lithium-responsive disorders in humans.

## Introduction

Since the initial discovery in 1949 that lithium possesses mood-stabilizing properties (Cade, 1949), the alkali metal has remained one of the most widely used medications for bipolar disorder (BPD). Over the years, various hypotheses have been proposed to explain the physiological effects of lithium. These stem mainly from lithium’s ability to inhibit, either directly or indirectly, particular enzymes such as glycogen synthase kinase 3β (GSK-3β; Klein and Melton, 1996), inositol monophosphatase (IMPase), and inositol 1-polyphosphate phosphatase (IPPase; Berridge et al., 1989; Shastry, 1997). By inhibiting these enzymes, lithium is thought to alter the signaling cascades in which they participate, ultimately influencing an array of physiological processes (Manji and Lenox, 1998; Phiel and Klein, 2001; Lenox and Wang, 2003; Machado-Vieira et al., 2009). However, the exact mechanisms by which lithium modifies neural function and improves pathophysiological behaviors are still not fully understood.

Studies using animal models suggest that the effects of lithium on the nervous system have common features across a range of diverse animal species. In particular, the fruit fly *Drosophila melanogaster* displays a neurobiological response to lithium resembling that observed in mammals. For instance, chronic treatment with lithium lengthens the free-running period of the circadian rhythm in both mice and fruit flies (Padiath et al., 2004; Dokucu et al., 2005; Iitaka et al., 2005). Likewise, lithium can protect against polyglutamine-induced neurotoxicity in *Drosophila* (Berger et al., 2005), as is observed in rodent models of Huntington’s disease (Wei et al., 2001; Senatorov et al., 2004). Furthermore, lithium treatment can rescue the behavioral deficits exhibited by the *Drosophila fragile X mental retardation 1* (*dfmr1*) mutant, as was observed in human fragile X patients and rodent models of the disease (McBride et al., 2005; Berry-Kravis et al., 2008; Yuskaitis et al., 2010). These findings indicate that studies in *Drosophila* can provide valuable insights into the basic mechanisms of lithium action on neural function and behavior.

*Shudderer* (*Shu*) was originally isolated more than 40 years ago, through an ethyl methanesulfonate mutational screen in *Drosophila* (Williamson, 1971), as an X chromosome–linked dominant mutation that causes sporadic jerks and defects in reactive climbing behavior (Williamson, 1982). Interestingly, Williamson reported that these behavioral phenotypes were significantly suppressed when adult *Shu* mutants were fed a diet supplemented with lithium salts (Williamson, 1982). This finding implied that the *Shu* mutation affects genes related to lithium-responsive neurobiological processes. To our knowledge, however, no research on *Shu* has followed since the original report in 1982, and this mutation remains to be characterized at both the molecular and functional levels. In the present study, we have identified *Shu* as a hypermorphic mutation in the *Drosophila* voltage-gated sodium (Na_v_) channel gene *paralytic* (*para*) and found that lithium also mitigates behavioral phenotypes of some other *para* mutants. A combination of molecular, pharmacological, electrophysiological, and behavioral analyses from our study suggests that lithium’s suppressive effect on *Shu* phenotypes is not due to acute actions of the drug but rather through long-term physiological adjustments to lithium treatment. Our results connecting lithium with Na_v_ channel dysfunction demonstrate that *Shu* and other Na_v_ channel mutants in *Drosophila* are valuable genetic tools for investigating the effects of lithium on the nervous system in the context of neurophysiology and behavior.

## Materials and Methods

### Fly stocks and culture conditions

Fly cultures were reared at 25°C in a 12-h light/dark cycle on a conventional cornmeal/glucose/yeast/agar medium supplemented with the mold inhibitor methyl 4-hydroxybenzoate (0.05%). Flies used for electrophysiological experiments were raised on a fly food formulation described previously (Frankel and Brousseau, 1968). The *Canton-S* strain (*CS*) was used as the wild-type control. *Shu* (Williamson, 1982) was obtained as *Shu*/*FM6* from Rodney Williamson (Beckman Research Institute of the City of Hope, Duarte, CA). The original *Shu* mutants were outcrossed to the *CS* line at least 26 times and balanced with *FM7*-*Actin-GFP*. *Shu*/*FM7*-*Actin-GFP* females were crossed to *CS*, and the resultant *Shu*/+ flies were used for most experiments in this study. The RNAi lines *w^1118^*; P[UAS-*para-RNAi*] (GD6131 and GD6132) and *w^1118^* control were obtained from the Vienna Drosophila Resource Center, Vienna. The following P-element lines were obtained from the Bloomington Drosophila Stock Center, Bloomington, IN: *y^1^ w^67c23^ P[EPgy2]Axs^EY00887^; y^1^ w^67c23^ P[EPgy2]CanA-14F^EY08594^; y^1^ w^67c23^ P[EPgy2]CG9902^EY05861^; y^1^ w^67c23^ P[EPgy2]Rbp2^EY00852^; y^1^ w^67c23^ P[EPgy2]CG4239^EY01983^*, *y^1^ w^67c23^ P[EPgy2]eas^EY01463^*, *y^1^ w^67c23^ P[EPgy2]CanA-14F^EY09066^*, and *y w P[GawB]NP6106*/*FM7c.* Other fly strains used in this study were obtained from the following sources: *para^bss1^* from Dr. Mark Tanouye (University of California, Berkeley, CA); *para^GEFS+^*, *para^DS^*, and their control from Dr. Diane O’Dowd (University of California, Irvine, CA).

### Mapping procedure

Meiotic recombination mapping of the *Shu* mutation was conducted using molecularly defined P-element insertion lines as described in Zhai et al. (2003). Briefly, a *white* (*w*) mutation was first introduced into the *Shu* chromosome (*w Shu*), which was balanced with *w FM7*. In the F1 generation, virgin female flies trans-heterozygous for *w Shu* and a P-element insertion (*w Shu*/*w P*) were crossed to *w* males. In the F2 generation, recombinants were identified as *Shu* mutants with orange eyes (*w Shu P*/*w* or *w P Shu*/*w*) or non-*Shu* flies with white eyes (*w*/*w*). The projected molecular position was calculated from the percentages of recombinants and the insertion sites of P-elements in base pairs between each combination of P-element pairs flanking either side of the projected lesion site established from earlier mapping (Zhai et al., 2003).

### Behavioral analyses

#### Shuddering

Newly eclosed virgin *Shu* and *CS* females were collected under CO_2_ anesthesia. To eliminate possible effects of downturned wings on shuddering assays, wings of the mutant and control flies were cut with microdissecting scissors (Ladd Research, Williston, VT). Fly cultures were kept and tested in an environmental chamber at 25°C and 60–70% humidity. Flies were individually placed into standard mating chambers (15-mm diameter, 3-mm depth) using a manual aspirator and allowed to acclimate for 5 min. At the end of 5 min, fly behavior was video recorded (DCR-PC300; Sony, Tokyo) for 2 min. *Shu* and wild-type flies were scored for the number of times that a strong convulsive episode flipped the fly onto its back. The scoring investigators were blinded to the experimental treatments. At least 30 flies were used for each treatment, genotype, and time point tested.

#### Reactive climbing

The reactive climbing assay was performed as previously described (Greene et al., 2003) using a countercurrent apparatus originally invented by Seymour Benzer for analyzing phototaxis behaviors (Benzer, 1967). Briefly, virgin females of each genotype were collected shortly after eclosion. To ensure that climbing was not affected by the downturned wing phenotype of *Shu* mutants, wings of the mutant and control flies were cut with microdissecting scissors. Flies were aged in groups of ∼20. At the time of the experiment, they were placed into one tube (tube 0), tapped to the bottom, and allowed 15 s to climb, at which point the flies that had climbed were transferred to the next tube. This process was repeated a total of five times. After the fifth trial, the flies in each tube (0–5) were counted. The climbing index was calculated using the following formula: CI = Σ(*N_i_* × *i*)/(5 × Σ*N_i_*), where *i* and *N_i_* represent the tube number (0–5) and the number of flies in the corresponding tube, respectively. At least five groups were tested for each genotype or treatment.

#### Video tracking locomotor analysis

Newly eclosed virgin females were collected and aged for 5 d. Flies were individually placed into standard mating chambers using a manual aspirator and allowed to acclimate for 5 min. At the end of 5 min, fly behavior was recorded using a web camera (Logicool Quickcam IM, Logitech, Fremont, CA). Images of individual flies were captured at 15 frames/s for 10 min and analyzed using pySolo (Gilestro and Cirelli, 2009) to track fly locomotion and compute *x*, *y* coordinates during every frame for a total of 9000 frames. The percentage of time a fly spent inside an inner circle (70% diameter of the chamber) during the 10-min observation period was calculated from its *x*, *y* coordinates.

#### Ether-induced shaking

Five-d-old flies of the indicated genotypes were introduced into a *Drosophila* etherizer (Science Kit & Boreal Laboratories, Tonawanda, NY) and exposed to a saturated dose of diethyl ether for 10 s. A drop of adhesive was then applied to the posterior dorsal thorax of each fly and fixed to a piece of plain white paper in a 35 × 10-mm Petri dish. Flies were then allowed to recover for 2–3 min before video recording using a Quickcam connect camera (Logitech) mounted on a Leica MZFLIII stereoscope (Leica Microsystems, Bannockburn, IL). Images were captured at 15 frames/s for 1 min and analyzed using pySolo to generate *x*, *y* data. Head movement was tracked by generating a mask (i.e., cropping out the entire image outside the selected area) and focusing on the anterior lateral region of the eye and the background. This area was selected based on initial trials, which indicated it to be the most consistent region in which to track head movement without picking up antennal motion. Leg movement was tracked by producing a mask selection encompassing the joint between the tibia and tarsus of the hind leg. Special care was taken to use video only where no other appendages or body parts entered the tracking mask during the 1-min recording. Velocities of both the head and leg movements between frames were calculated using Microsoft Excel.

#### Heat-induced seizure

Heat-induced seizure assay was performed as described in Sun et al. (2012) using 5-d-old flies. Individual flies were isolated in a glass vial (15 × 45 mm) and acclimated for 15–30 min. The vials were submerged in a water bath set at either 37°C or 40°C for 2 min. At 5-s intervals, the status of individual flies was determined to be either seizing or not seizing, and the proportion of flies seizing at each time point was calculated. Heat-induced seizures were defined as a period of leg twitches, failure to maintain standing, wing flapping, or abdominal curling (Sun et al., 2012).

#### Heat-induced paralysis

Individual flies were isolated in a glass vial (15 × 45 mm) and acclimated for 10–15 min. Vials were submerged in a water bath set at 34°C and monitored for paralysis. Paralysis was defined as cessation of movement and/or loss of posture.

#### Bang sensitivity recovery assay

Individual flies were isolated in a glass vial (15 × 45 mm) and acclimated for 15–30 min. Flies were subjected to 10 s of mechanical shock delivered by a vortex at maximum intensity. The time to recover, defined as a return to a standing position, was scored for each fly (Marley and Baines, 2011).

### Scoring of morphological defects

Male and female *Shu* mutants were collected shortly after eclosion and scored 24 h later as either defective (i.e., downturned wings or indented thorax) or normal (wild-type wing posture/thorax).

### Lifespan assay

Newly eclosed *Shu* (*Shu*/+) and *CS* (+/+) females were collected under CO_2_ anesthesia shortly after eclosion and kept in groups of ∼10 or 20 in regular or lithium-containing food vials at 25°C, 60–70% humidity. Flies were transferred to new vials every 3 d, and survivors were scored every day.

### Drug treatment

Lithium chloride (LiCl; Sigma-Aldrich, St. Louis, MO) was dissolved in water to generate 1-, 0.5-, and 0.25-m stock solutions, which were added to the standard fly food in a 1:10 dilution to produce the final concentrations used in these studies. Newly eclosed flies were transferred to vials with LiCl-containing food and aged for 1–5 d before experiments were performed. For the experiments to evaluate the effects of inhibitors for GSK-3 or IMP, larvae were raised in the standard food containing 20 μm AR-A014418 (Sigma-Aldrich) or 0.5 mm L-690330 (Tocris, Bristol, UK) at 25°C. AR-A014418 and L-690330 were initially dissolved in DMSO and water to generate 20- and 0.5-m stock solutions, respectively. The eclosed adult flies were scored for their morphological phenotype and continued to be grown on the drug-containing food for 5–6 d before being assessed for locomotor activity.

### Measurement of internal lithium

Twenty virgin female flies were placed in a vial with food containing 25, 50, or 100 mm LiCl for 5 d and were homogenized in 350 μl of 1× PBS (pH 7.4). The homogenate was centrifuged at 15,000 rpm for 15 min and filtered through a Nanosep spin filter cartridge (0.2-μm pore size; Pal Corp., East Hills, NY). The supernatant was subjected to lithium analysis performed by the University of Iowa Hospitals and Clinics Pathology Laboratory using spectrophotometry with the Infinity lithium single liquid stable reagent (Thermo Fisher Scientific, Waltham, MA).

### Molecular biology

#### Genomic DNA isolation and sequencing

Genomic DNA was isolated from heads of wild-type or *Shu* males and used as a template for PCR amplifications. Based on FlyBase sequence information, primer sets were designed to amplify *para* exons. PCR amplifications were carried out using proofreading DNA polymerases as described in our previous study (Kasuya et al., 2009a). The PCR products were cloned into the pCR2.1 TA cloning vector (Invitrogen, Grand Island, NY) for sequencing analyses. Resulting exon sequences were compared between those of wild-type and *Shu* mutant flies to identify mutations. At least five clones from one PCR amplification were sequenced to confirm that the identified mutation was not the result of a PCR error.

#### Construction of pUAS-para plasmids

Total RNA was extracted from heads of wild-type or *Shu* males and used to generate complementary DNA (cDNA) with the Superscript III reverse transcriptase kit (Invitrogen). The cDNA was used as a template to amplify a 461-bp Blp1 fragment with the primers Na_v_1.1 BlpI, forward 5′-ATT TCC GAT CTT AGC CGG TG-3′ and reverse 5′-ACA GAT ACG CGT TAC CTA CAT GAT C-3′, respectively. The amplified fragment completely covered *para* exons 24–26 and partially covered exons 23 and 27. The *Shu* mutation resides in exon 24. The PCR products from both wild-type and *Shu* mutant cDNA were then purified, digested with Blp1, and cloned into pGH19-Na_v_1.1 plasmid kindly provided by Mark Tanouye (Parker et al., 2011), at Blp1 sites to replace the Blp1 fragment. Na_v_1.1 cDNAs with or without the *Shu* mutation were excised from the pGH19 vector using KpnI and XbaI and subcloned into the pUAST vector using the same restriction enzymes. The entire *para* cDNA was sequenced to confirm that no nonsynonymous nucleotide substitutions other than the *Shu* mutation (see Results) were present compared with Na_v_1.1.

### Adult flight muscle electrophysiology

As described previously (Engel and Wu, 1992; Lee and Wu, 2002; Iyengar and Wu, 2014), adult flies were briefly anesthetized on ice, tethered to a tungsten wire using cyanoacrylate glue, and allowed to rest for 30 min. For recordings of flight-muscle activity, an electrolytically sharpened tungsten electrode was inserted into the top dorsal longitudinal muscle (DLMa), and a similar reference electrode was placed into the abdomen. DLM action potentials (Ca^2+^ spikes) were amplified by an AC amplifier (bandwidth 10 Hz to 20 kHz, Model 1800, AM Systems, Sequim, WA) and digitized at 20 kHz by a USB 6210 DAQ card (National Instruments, Austin, TX) controlled by a custom script written in LabVIEW 8.6 (National Instruments). We examined the properties of the giant-fiber (GF) pathway as well as seizure discharges in DLMs triggered by electroconvulsive stimulation (ECS) across the brain. In addition, nonflight spontaneous DLM activity was monitored.

The two parameters of the GF pathway characterized were the DLM response latency and following frequency. The GF pathway was stimulated by 0.1-ms pulses at about 30 V generated by an isolated pulse stimulator (AM Systems Model 2100) delivered via tungsten electrodes inserted into each eye. In this study, DLM response latency was determined to be the time between GF stimulation and the time at which DLM spike reached its half-maximum height. The ability of the GF pathway to follow high-frequency stimulation was assessed by delivering a series of 10-pulse trains at increasing frequencies from 20 to 200 Hz, at 20-Hz increments, with 5 s between stimulus trains. The number of GF responses successfully recruited in each stimulus train was recorded, and the interpolated stimulus frequency at which five responses could be recruited indicated the 50% success following frequency (FF_50_).

The ECS protocol used to trigger DLM seizure discharges (Lee and Wu, 2002; Iyengar and Wu, 2014) consisted of a 2-s train of high-frequency pulses (0.1 ms, 200 Hz), with voltages ranging from 30 to 80 V as specified. To avoid refractoriness, flies were allowed to rest at least 10 min between ECS trains. ECS typically evokes a seizure discharge repertoire characteristic to different genotypes, including wild-type (Lee and Wu, 2002, 2006). In this study, we confined our analyses to the period immediately after ECS, which consists of a high-frequency burst of DLM spikes discharge corresponding with behavioral spasms (Pavlidis and Tanouye, 1995; Lee and Wu, 2002; Iyengar and Wu, 2014). In wild-type flies, this initial seizure discharge usually lasted ∼1 s, whereas it often lasts more than 10 s in *Shu* (Fig. 2*D*). Thus, our spike counts per trial included a period of 8 s, even though in the wild-type, the seizure discharge seldom persisted more than 1 s. We considered a burst activity beyond five spikes time-locked to ECS to indicate above-threshold activity. This criterion effectively differentiates near-threshold seizure discharges from sporadic background spontaneous firing often observed in a tethered fly (Engel and Wu, 1992; Lee and Wu, 2006).

### Larval neuromuscular junction electrophysiology

Preparation of postfeeding wandering third instar larvae and recordings of excitatory junctional potentials (EJPs) and nerve action potentials were carried out as described previously (Ueda and Wu, 2006; Lee et al., 2008). Briefly, dissections were performed in Ca^2+^-free HL3 saline (in mm): 70 NaCl, 5 KCl, 20 MgCl_2_, 10 NaHCO_3_, 5 trehalose, 115 sucrose, and 5 HEPES, at pH 7.2 (Stewart et al., 1994), and EJPs and nerve action potentials were recorded in the modified HL3.1 saline (with MgCl_2_ reduced to 4 mm and 0.2 mm CaCl_2_ added; Feng et al., 2004). The segmental nerve was severed from the ventral ganglion, and stimuli were delivered through the cut end with a suction electrode (10-μm internal diameter). The stimulation voltage was adjusted to twice the threshold to ensure action potential initiation. For EJP recordings, intracellular glass microelectrodes were filled with 3 m KCl and had a resistance of ∼60 MΩ. Signals were recorded with a DC pre-amplifier (model M701 microprobe system, World Precision Instruments, Sarasota, FL). Compound action potentials were recorded from the segmental nerve *en passant* with a suction pipette (Wu et al., 1978). Data were digitized and analyzed on a PC.

### Microarray analysis

Microarray analysis was performed as previously described (Kasuya et al., 2009b). One-d-old female flies were kept in a vial containing the regular cornmeal-based food with or without 50 mm LiCl for 24 h. Their heads were cut on a dry ice block and stored at –80°C until use. Total RNA was extracted from ∼75–100 fly heads using TRIzol Reagent (Invitrogen) followed by an RNeasy (Qiagen, Valencia, CA) cleanup step and DNase I digestion. The RNA was resuspended in diethylpyrocarbonate-treated water and subjected to microarray analysis. Three independent RNA samples were prepared and analyzed for each treatment group. Microarray experiments were carried out at the Translational Genomics Research Institute (Phoenix, AZ) using Affymetrix *Drosophila* Genome 2.0 Arrays (Affymetrix, Santa Clara, CA). Each chip is composed of 18,800 probe sets representing more than 18,500 transcripts. Image data were quantified by using the Genechip-operating software Affymetrix GCOS v1.4. Gene expression data were analyzed using GeneSpring software. We focused on genes that were detected in all three replicates for at least one of the two conditions in comparison. The comparisons were made between signals for *CS* and *Shu*/*+* with or without lithium treatment using Welch’s *t*-test (Kasuya et al., 2009b). Comparisons were also made using one-way ANOVA, and Bonferroni multiple-comparison corrections were applied to obtain the false discovery rate. Genes were annotated and biological processes were analyzed using Database for Annotation, Visualization, and Integrated Discovery (DAVID) v6.7 (https://david.ncifcrf.gov/; Huang et al., 2009).

### Reverse-transcription PCR analysis

For semiquantitative and real-time reverse-transcription (RT)-PCR, RNA was extracted using the methods described above, and single-strand cDNA libraries were synthesized with DNase I-treated RNA using Superscript III reverse transcriptase kit (Invitrogen) or, in the real-time experiments, iScript cDNA Synthesis Kit (Bio-Rad Laboratories, Hercules, CA). For semiquantitative RT-PCR, conditions for PCR reactions were optimized for each gene such that the endpoint of each PCR reaction was in the linear range of amplifications. Transcript-level quantification analyses were measured by analyzing pixel intensity of the bands using Image J (NIH). For quantitative real-time RT-PCR, cDNA samples were analyzed using a CFX96 Real-Time System (Bio-Rad Laboratories), with each 20 μl reaction containing 10 μl of SsoAdvanced Universal SYBR Green Mastermix (Bio-Rad Laboratories), 2 ng of cDNA, and RT primers designed using PrimerQuest (Integrated DNA Technologies, Coralville, IA) at a final concentration of 300 nM. Before RT-PCR experiments, primer efficiencies were determined from dilution curves (1:10) using the formula: *E* = 10^–1^/slope (Pfaffl, 2001). Fold-change was determined using the ΔΔCt method (Livak and Schmittgen, 2001). Primers used in the RT-PCR experiments are listed in Table 7.

### Statistical analysis

For behavioral experiments, statistical comparisons between two groups were performed using a two-tailed Student’s *t*-test assuming unequal variance or, in the case of non–normally distributed data, the Mann–Whitney *U*-test was used. Statistical significance between multiple groups displaying a normal distribution was determined using one-way ANOVA with Bonferroni-corrected *t*-test comparisons between control and treatment groups post hoc. For those data exhibiting nonnormal distributions, Kruskal–Wallis one-way ANOVA on ranks was performed. Comparisons between groups or groups versus a control were calculated using Dunn’s method post hoc. Data not conforming to a normal distribution are represented as box plots in most cases. The log-rank Kaplan–Meier survival test was applied to analyze survival time. Statistical analyses were performed using SigmaStat for Windows Version 3.11 (Systat Software, Point Richmond, CA). Differences between mean or median values were considered significant at *p* < 0.05. The microarray gene expression data were analyzed using GeneSpring software (Agilent Technologies, Santa Clara, CA). The comparisons were made between genotypes (i.e., +/+ or *Shu*/+) or drug treatments (i.e., with or without LiCl). Lists were filtered for the genes that were present in all three independent samples of at least one of the two genotypes.

## Results

### Outcrossed *Shu* mutants exhibit morphological and behavioral abnormalities

In the original study by Williamson (1982), phenotypes of *Shu* mutants were examined using *Shu/FM6* female flies, where *FM6* is a balancer X chromosome with multiple inversions and marker mutations. It was therefore possible that genetic aberrations in the *FM6* balancer chromosome contributed to the previously reported dominant phenotypes of *Shu*. To rule out this possibility, the following phenotypic analyses were carried out with the progeny of crosses between control males and *Shu* females with the X chromosome balancer (i.e., *Shu*/+ and *Shu/Y*). To minimize potential effects of unidentified genetic variations in the original *Shu* genome, we used a *Shu* mutant stock that had been backcrossed to *CS* for at least 26 generations. Outcrossed *Shu* males (*Shu/Y*) survived for only a few days after eclosion and were too sluggish to perform behavioral tasks. Under our standard rearing conditions, *Shu* males displayed severe defects in courtship behavior and rarely copulated. Consequently, homozygous adult *Shu* females (*Shu*/*Shu*) were not normally found in *Shu* mutant stocks. Our reassessment revealed morphological and behavioral defects in *Shu* mutants, some of which had been reported for the original *Shu*/*FM6* stock in Williamson (1982); others are described for the first time in this study.

**Figure 1.**
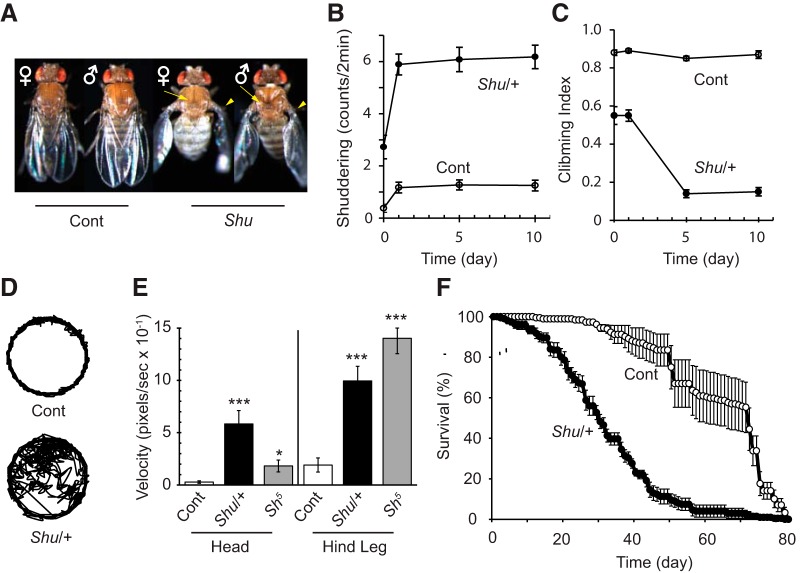
*Shu* mutants exhibit abnormalities in morphology, behavior, and lifespan. ***A***, *Shu* males (*Shu/Y*) and females (*Shu*/+) often display downturned wings (arrow head) and an indented thorax (arrow), which are not observed in wild-type flies (Cont). ***B***, 0- to 10-d-old *Shu* (*Shu*/*+*) and wild-type (Cont) female flies were scored for the number of times in 2 min that a strong convulsive episode resulted in the fly being thrown onto its back. Wings of the mutant and control flies were removed shortly after eclosion to eliminate possible effects of downturned wings of *Shu* mutants on shuddering behavior. Data are mean ± SEM. *n* ≥ 30. ***C***, The general activity and motor coordination of *Shu* mutants were assessed by a reactive climbing assay using a countercurrent apparatus. 0- to 10-d-old wild-type (Cont) and *Shu* (*Shu*/+) females were used. To ensure that the climbing defect was not simply due to the downturned wing phenotypes of the fly, wings of the mutant and control flies were removed shortly after eclosion. Data are mean ± SEM for 10 groups of 20 flies. ***D***, Representative locomotion traces of wild-type (Cont) and *Shu* (*Shu*/+) females in a circular chamber. Compared with wild-type flies, which travel mostly along the edge of the chamber, *Shu* mutants spent significantly longer times in the center of the chamber. ***E***, Head and hind leg movements under ether anesthesia were video recorded at 15 frames/s, and their velocities (pixels/frame) in wild-type (Cont), *Shu* (*Shu*/+), and *Sh^5^* females are shown as mean ± SEM. Data for head and leg movements were analyzed using Kruskal–Wallis one-way ANOVA on ranks followed by Dunn’s test, and one-way ANOVA followed by Bonferroni test, respectively. The statistical significance of differences between wild-type (Cont) and each genotype (*Shu*/+ or *Sh^5^*) is shown. *p < 0.05; ***p < 0.001. *n* = 11. ***F***, Survival of *CS* (Cont) and *Shu* (*Shu*/+) females under standard conditions at 25°C, 60–70% humidity. Flies were kept in groups of ∼20. Data are presented as the daily average percentage of surviving flies in each vial with SEM. *n* = 180 and 176 for *CS* and *Shu*, respectively. The median survival times were 72 and 30 d for wild-type and *Shu*. Two survival curves showed a statistically significant difference (*p* < 0.001, log-rank Kaplan–Meier survival analysis).

*Shu* males (*Shu/Y*) and heterozygous females (*Shu*/+, hereafter referred to as *Shu* or *Shu* females unless otherwise stated) were found to exhibit an abnormal wing posture (downturned wings) and a cuticular defect on the back (indented thorax), neither of which was observed in wild-type flies (Fig. 1*A*). These morphological phenotypes manifested shortly after eclosion and at high penetrance in both sexes. In *Shu* males, 88% and 69% exhibited downturned wings and an indented thorax, respectively (*n* = 272), whereas in *Shu* females 73% and 65% displayed the defects (*n* = 683). Similar morphological abnormalities have been reported in *Shaker* (*Sh*) mutants harboring a second genetic lesion in either *ether-a-go-go* (*eag*; Ganetzky and Wu, 1983; Engel and Wu, 1992) or *receptor oscillation A* (*rosA*; Wu and Wong, 1977) also known as *inebriated* (*ine*; Stern and Ganetzky, 1992), or when combined with a *para* duplication (Stern et al., 1990). *Sh* and *eag* encode the α-subunit of voltage-gated potassium channels K_v_1 (Kamb et al., 1987; Papazian et al., 1987; Pongs et al., 1988) and K_v_10 (Warmke et al., 1991), respectively, whereas *rosA*/*ine* encodes the neurotransmitter/osmolyte transporter (Burg et al., 1996; Soehnge et al., 1996). Double mutants for the gene *quiver* (*qvr*; Humphreys et al., 1996) and either *eag* or *Hyperkinetic* (*Hk*; Kaplan and Trout, 1969) show similar defects in wing posture and thoracic morphology (Wang et al., 2000). *Hk* encodes the auxiliary β-subunit for the Sh K_v_1 channel (Chouinard et al., 1995), and mutations of *Hk* and *qvr* modify the *Sh* K current (Wang and Wu, 1996; Yao and Wu, 1999; Wang and Wu, 2010). These morphological abnormalities are thought to be caused by an increase in neuronal excitability that leads to hypercontraction of the relevant thoracic muscles (Engel and Wu, 1992; Huang and Stern, 2002).

As reported by Williamson (1982), *Shu* mutants displayed sporadic, spontaneous tremors or “shuddering.” This behavioral abnormality was nearly absent during the first 8 h after eclosion. However, 24 h later, the frequency of severe shuddering episodes (resulting in the fly being thrown onto its back) increased significantly and remained at this level throughout the 10-d period of observation (Fig. 1*B*). In contrast, wild-type flies displayed these types of episodes only rarely when first introduced into the observation chamber. We also performed a reactive climbing assay using a countercurrent apparatus (Benzer, 1967) to assess the general activity and motor coordination of *Shu* mutants. Defects in this behavior were clearly evident in *Shu* mutants soon after eclosion and further deteriorated in an age-dependent manner (Fig. 1*C*).

In an effort to better quantify the behavioral abnormalities of *Shu* mutants (e.g., jerking, twitching, and uncoordinated locomotion), we also analyzed their movements using an automated system originally developed for *Drosophila* sleep analysis (Gilestro and Cirelli, 2009). When placed in a circular chamber, wild-type flies spent most of their time along the periphery (Besson and Martin, 2005), resulting in tracking patterns resembling a circle. In contrast, the uncoordinated movements and spontaneous jerking of *Shu* mutants led to their increased presence in the center part of the chambers, as represented by a typical tracking trace (Fig. 1*D*). During the 10-min recording period, *Shu* mutants spent a significantly longer time in the center than wild-type flies. Typical median percentages of time spent in the center of the chamber were 4.8% for wild-type flies (*n* = 63) and 39.5% (*n* = 53) for *Shu* mutants (*P* < 0.001).

### *Shu* mutants display an ether-induced shaking phenotype and reduced lifespan

*Shu* mutants were reported to exhibit leg-shaking behavior under ether anesthesia (Williamson, 1982). This behavioral phenotype has previously been observed in several other mutants with enhanced neuronal excitability, such as the aforementioned *Sh*, *eag*, *Hk*, and *qvr* mutant strains (Kaplan and Trout, 1969; Wang et al., 2000). To analyze this behavioral phenotype in a semi-quantitative manner, we recorded ether-induced movements of the head and hind leg using a video-tracking system (see Materials and Methods). *Shu* mutants vigorously moved the head as well as the legs under ether anesthesia, resulting in a drastic increase in the velocities of their movements compared with wild-type flies (Fig. 1*D*). The ether-induced shaking phenotype of *Shu* was similar to, but distinct from, that of *Sh^5^*, a neomorphic mutant allele of *Sh* (Salkoff and Wyman, 1983; Haugland and Wu, 1990), in that *Shu* had a tendency to waggle the head more extensively than *Sh* (Fig. 1*E*), and that the leg movements of *Shu* were sporadic, whereas those of *Sh* were rather continuous. Moreover, legs severed from ether-anesthetized *Sh* continued to shake (Ganetzky and Wu, 1985) whereas those severed from *Shu* did not, suggesting that *Shu*’s ether-induced leg shaking phenotype likely has a central origin.

Shortened lifespan is a common feature of *Drosophila* mutants that display ether-induced leg-shaking, such as *Sh*, *Hk*, and *qvr* (Trout and Kaplan, 1970; Koh et al., 2008; Bushey et al., 2010). Similarly, the median survival time of *Shu* mutants was significantly reduced (30 d, *n* = 176) compared to that of wild-type flies (72 d, *n* = 180; *p* < 0.001, Fig. 1*F*).

### DLMs of *Shu* mutants exhibit drastically enhanced spontaneous activity and ECS seizure discharges

**Figure 2.**
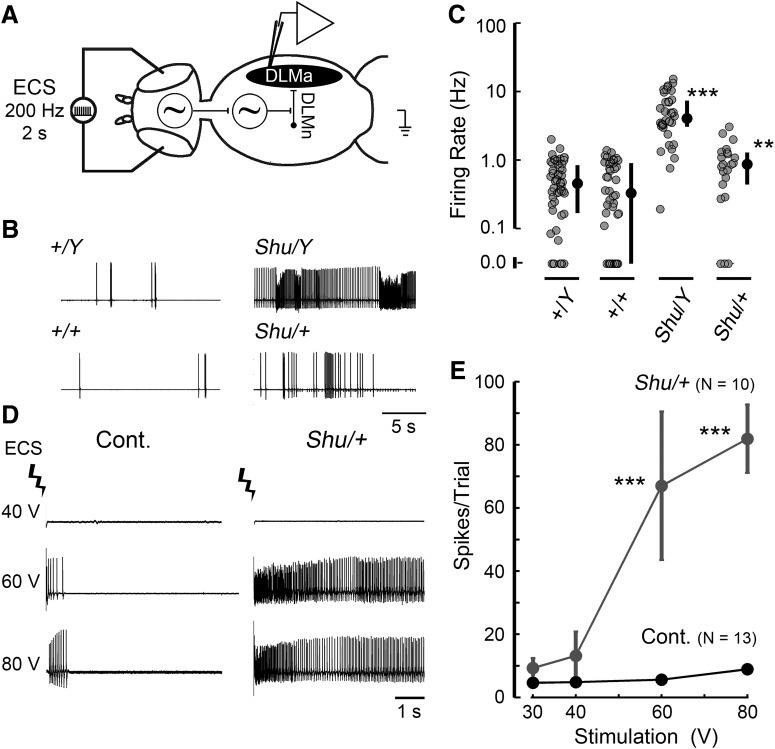
*Shu* mutants display hyperexcitable seizure-like discharges in the electrical output of an identified motor neuron. ***A***, The dorsal longitudinal motor neuron (DLMn) receives input from a number of pattern generators, and its activity may be monitored via spiking activity in the dorsal longitudinal muscle (DLM). ***B***, Example traces of spontaneous activity in *Shu* males (*Shu*/*Y*) and females (*Shu*/*+*; right) compared with wild-type control individuals (left). ***C***, Scatterplot of spontaneous firing rates in control (+/*Y*, +/+) and *Shu* (*Shu*/*Y, Shu*/*+*) populations. Each gray circle represents a 1-min recording of activity, black circles (right) represent the median, and vertical bars represent the 25th–75th percentile interval (*n* > 10 individuals for each group). **p < 0.01; ***p < 0.001, Kruskal–Wallis ANOVA, rank-sum post hoc test. ***D***, DLM firing, monitored as readout of stereotypic seizure-like discharges triggered by high-frequency, high-voltage electroconvulsive stimulation across the brain (ECS, see Materials and Methods). Sample ECS-triggered discharges in control (Cont) and *Shu*/+ individuals are displayed. ***E***, Plot of ECS voltage and number of spikes per discharge, a measure of seizure intensity. *Shu*/+ individuals display significantly more intense seizure discharges at 60 and 80 V compared with control (Cont) flies. ***p < 0.001, Kruskal–Wallis ANOVA, rank-sum post hoc test.

The behavioral and morphological phenotypes of *Shu* mutants suggest that the excitability of their motor circuits is enhanced. The dorsal longitudinal muscles are particularly amenable to examination of changes in motor circuit function, because they serve as outputs of the motor neurons that integrate inputs, including those from the flight pattern generator (Harcombe and Wyman, 1977), and the GF circuit mediating the jump-and-flight escape reflex (Tanouye and Wyman, 1980; Fig. 2A). DLMs’ large size and isometric contraction enable prolonged monitoring of their action potentials (Ca^2+^ spikes). Importantly, this system has revealed alterations in basic physiological properties caused by a number of hypo- and hyper-excitable mutations (Siddiqi and Benzer, 1976; Elkins et al., 1986; Engel and Wu, 1992, 1998; Iyengar and Wu, 2014).

Increased spontaneous nonflight DLM activity is a basic phenotype associated with hyperexcitable mutants, including the double potassium channel mutant, *eag Sh* (Engel and Wu, 1992). Figure 2 illustrates this spontaneous activity in *Shu* mutant DLMs. We found that heterozygous *Shu* females displayed a significantly increased spike frequency compared with wild-type females (0.99 ± 0.24 Hz vs. 0.50 ± 0.10 Hz, *p* < 0.01; Fig. 2*B*, *C*). In hemizygous *Shu* males, this phenotype was much more striking compared with wild-type males (5.56 ± 0.93 Hz vs. 0.53 ± 0.08 Hz, *p* < 0.001) and even greater than that previously observed in *eag Sh* double mutants (∼3.5 Hz; Engel and Wu, 1992). In contrast to these findings in spontaneous activities, analysis of the GF pathway–mediated responses to electrical stimulation across the brain revealed relatively mild effects of the *Shu* mutation. We examined both male and female *Shu* individuals for DLM spike initiation latency and the interpolated stimulus frequency at which 50% DLM responses failed (FF_50_). We found only a slightly retarded DLM spike latency in *Shu* males and a modest increase in FF_50_ in *Shu* females compared with wild-type flies (Table 1).

**Table 1.** Electrophysiological properties of the GF circuit in wild-type flies and *Shu* mutants.

Property	+/+	*Shu*/+	+/*Y*	*Shu*/*Y*
Latency, ms	1.67 ± 0.04 (16)	1.72 ± 0.05 (9)	1.52 ± 0.04 (21)	1.98 ± 0.13* (7)
FF_50_, Hz	168.0 ± 3.56 (13)	181.8 ± 3.90* (14)	187.4 ± 2.63 (24)	183.7 ± 3.23 (7)

*p < 0.05; Student’s *t*-test compared with wild-type counterparts. Sample numbers are indicated in parentheses.

The same recording configuration was used to examine the ECS-triggered stereotypic seizure-like discharge pattern. High-intensity, high-frequency ECS (up to 80 V, 200 Hz) across the brain evokes seizure activities characteristic of wild-type and a variety of mutant flies (Pavlidis and Tanouye, 1995; Lee and Wu, 2002, 2006; Ueda et al., 2008; Parker et al., 2011; Ehaideb et al., 2014). We quantified the spike patterns immediately after the ECS (initial discharge; Lee and Wu, 2002) and found that in *Shu* females’ ECS-triggered discharges were far more intense than those of wild-type females in both number of spikes per discharge (Fig. 2*D*, *E*) and duration of spike discharges. This duration was consistently >5 s in *Shu* females (8 of 10 flies), which was rarely observed in wild-type flies. However, the ECS intensity threshold did not differ significantly between *Shu* and wild-type females (∼60 V; see Materials and Methods for details).

### *Shu* maps to the *Drosophila* voltage-gated sodium channel gene *paralytic*

**Figure 3.**
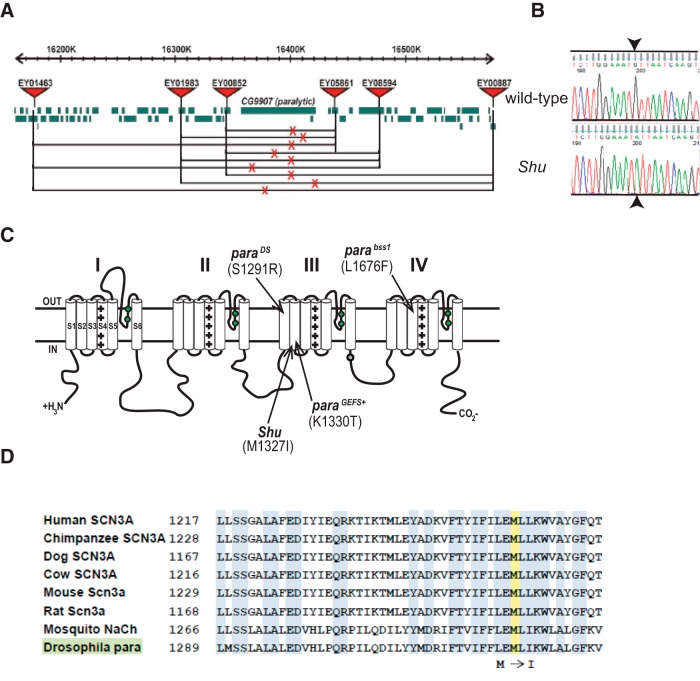
*Shu* maps to the voltage-gated sodium channel gene *paralytic.* ***A***, Mapping positions of the *Shu* mutation on the X chromosome. Red triangles and horizontal lines represent pairs of molecularly defined P-element insertions used to estimate the *Shu* mutation site; red X’s indicate the mutation sites deduced from the recombination rates between the corresponding P-element insertion and *Shu*. The estimated sites all reside within the *para* locus (CG9907). Boxes designate annotated genes near the *para* locus (based on FlyBase). ***B***, DNA sequencing chromatogram identifying a G-to-A transition mutation (arrowheads) in the *Shu* genome at the position corresponding to the nucleotide 4249 in the *para-RE* cDNA (FlyBase). This mutation results in a methionine-to-isoleucine substitution at the amino acid position 1327. ***C***, Schematic structural diagram of a *Drosophila* voltage-gated sodium channel. Arrow indicates the *Shu* mutation in the transmembrane segment S2 in homology domain III. The *para^GEFS+^* mutation K1330T, which corresponds to a *SCN1A* mutation K1270T causing GEFS+ in humans (Sun et al., 2012), lies three codons away from that of *Shu*. Also shown are the *para^DS^* mutation S1291R and the *para^bss1^* mutation L1676F. ***D***, Amino acid sequence alignment of Na_v_ channels of different animal species. Note that the methionine residue, which is mutated to isoleucine in *Shu*, is present in all Na_v_ channels.

The *Shu* mutation was previously mapped to the X chromosome, at the genetic map position 55.1 with respect to the *vermilion* and *forked* loci (Williamson, 1982), leaving us with a rough estimate of where the lesion might be located. We undertook a recombination-based mapping approach using molecularly defined P-element insertions to narrow down the chromosomal position of the *Shu* mutation (Zhai et al., 2003). To do this we recombined an eye color marker, the *white* mutation (*w*), into the *Shu*-carrying chromosome and scored nearly 50,000 F2 flies for the recombination events between *Shu* mutation and six nearby P-element insertions containing the *w^+^* mini gene (Table 2). Calculation of the recombination rates revealed that the P-element insertions *EY05861* and *EY00852,* nearest the gene *para* (*CG9907*), provided the lowest recombination rates (0.18 and 0.22%, respectively), whereas insertions more distant in either direction from cytological region 14E resulted in higher rates of recombination (Table 2). Based on the molecular distance between the defined P-element insertion sites and the recombination rates between these markers and the *Shu* mutation, the mutation site was estimated between the insertion sites of two particular P-elements used in our screen: *P[EPgy2]CG9902^EY05861^* and *P[EPgy2]Rbp2^EY00852^*. All combinations pairing one insertion distal, and one proximal, to cytological region 14E resulted in an estimated *Shu* mutation site in the 72-kb *para* locus (Fig. 3*A*), which encodes for the *Drosophila* voltage-gated sodium channel (Loughney et al., 1989).

**Table 2.** Recombination rates between *Shu* and P-element markers.

P-element (inserted in/near)	Total flies scored	Parental genotype	Recombinant genotype	Recombination rate
*EY00887 (Axs)*	12,881	12,780	101	0.78
*EY0859 (CanA-14F)*	6763	6730	33	0.49
*EY05861 (CG9902)*	6723	6711	12	0.18
*EY00852 (rbp2)*	8626	8607	19	0.22
*EY01983 (CG4239)*	4923	4888	35	0.71
*EY01463 (eas)*	7866	7798	68	0.86

### *Shu* carries a missense mutation in the *paralytic* coding region

Because our recombination mapping indicated that the *Shu* mutation is located within the *para* locus, we sequenced all 31 *para* coding exons using genomic DNA from extensively backcrossed (40 generations) hemizygous *Shu* males. Our results revealed a single nucleotide change in *Shu*, a G-to-A transition in *para* exon 24 (Fig. 3*B*) that results in the substitution of an isoleucine for a methionine at amino acid 1327 (based on *para-*PE, FlyBase). We confirmed that this mutation was also present in the genomic DNA isolated from the original *Shu* stock obtained from R. Williamson, but not in the wild-type strain used to isogenize the mutant stock. M1327I lies in the predicted transmembrane segment S2, within homology domain III of the sodium channel (Fig. 3*C*). Although various splicing variants are produced from the *para* locus, the exon containing the *Shu* mutation is constitutively spliced (Olson et al., 2008; Lin et al., 2009). Therefore, this amino acid replacement must be present in all Na_v_ isoforms in *Shu* mutants. In addition, the methionine mutated in *Shu* represents a highly conserved residue located in the Na_v_ channel α subunit proteins of evolutionarily diverse animal species (Fig. 3*D*), including all nine human Na_v_ channel α subunit isoforms (Yu and Catterall, 2003), strongly indicating that it is important for Na_v_ channel function.

### Targeted expression of the *Shu* Na_v_ channel partially phenocopies *Shu* phenotypes

**Figure 4.**
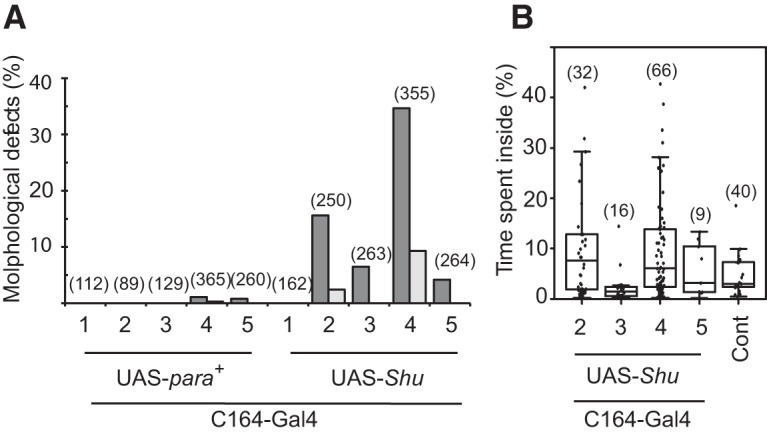
Targeted expression of the *Shu* Na_v_ channel partially phenocopies *Shu* phenotypes. ***A***, Effects of expression of the wild-type (UAS-*para^+^*) and *Shu* (UAS-*Shu*) Na_v_ channels using C164-Gal4 on morphological phenotypes. Indicated are percentages of flies with downturned wings (closed) or an indented thorax (open). The progeny of four of five independent UAS-*Shu* lines displayed downturned wings or an indented thorax, whereas the progeny of UAS-*para^+^* lines rarely displayed the morphological defects. ***B***, Effects of expression of the *Shu* Na_v_ channels on behavioral phenotypes. Percentages of time spent in the center of the chamber are shown for the progeny of C164-Gal4 and UAS-*Shu* lines. Sample numbers (*n*) are indicated in parentheses.

To directly examine whether the identified *Shu* mutation in *para* is sufficient to cause the mutant phenotypes, we generated multiple fly lines carrying a *para* cDNA transgene with or without the *Shu* mutation (UAS-*Shu* and UAS-*para^+^*). When five independent UAS-*Shu* or UAS-*para^+^* lines were crossed to the pan-neuronal driver *elav-*Gal4, the progeny did not show obvious morphological or behavioral abnormalities resembling those of *Shu* mutants. However, when UAS-*Shu* was crossed with C164-Gal4, an enhancer trap Gal4 line that drives gene expression widely in the nervous system including in motor neurons (Torroja et al., 1999; Jepson et al., 2012), a significant portion of the resulting progeny showed morphological defects similar to those seen in *Shu* mutants (Fig. 4*A*). Namely, 24 and 39% of the progeny of UAS-*Shu* lines 2 and 4, respectively, exhibited downturned wings. Such defects were rarely seen in any progeny of UAS-*para^+^* lines. The progeny of C164-Gal4 and UAS-*Shu* with the morphological abnormalities were further subjected to behavioral analysis. They were found to display spontaneous jerking and twitching, similar to that observed in *Shu* mutants. In general, their locomotion was disorganized, as demonstrated by the video tracking method (Fig. 4*B*). The severity of the phenotype varied considerably among individuals, and only the progeny of UAS-*Shu* line 2 showed a statistical difference in this behavioral parameter (i.e., time spent in the center) compared with wild-type flies when the Mann–Whitney *U*-test was applied (*p* < 0.001). However, the progeny of UAS-*Shu* line 4 also exhibited abnormal behavior based on the observation that 14% of the tested progeny of UAS-*Shu* line 4 (9 of 66) spent >20% of their time in the center, whereas no control flies ever passed this time threshold (0 of 40). These results demonstrated that targeted expression of the *Shu* Na_v_ channel can mimic, at least partially, the morphological and behavioral abnormalities observed in *Shu* mutants.

### Functional interactions between *Shu* and other *para* mutant alleles

To infer the functional nature of the *Shu* mutation in *para*, we examined genetic interactions between *Shu* and other *para* mutant alleles, *para^ts1^*, *para^ts115^*, *para^bss1^*, and *para^GEFS+^*. Previous studies demonstrated that *para^ts1^* and *para^ts115^* exhibit recessive, temperature-induced paralysis (Suzuki et al., 1971; Song and Tanouye, 2007), whereas *para^bss1^* shows semidominant bang-sensitive paralysis (Parker et al., 2011). Generalized epilepsy with febrile seizures plus (GEFS+) is a common childhood-onset genetic epilepsy syndrome, often caused by a mutation in the human Na_v_ channel gene *SCN1A* (Scheffer and Berkovic, 1997). *para^GEFS+^* was generated by introducing a *para* mutation (K1330T) mimicking the GEFS+-causing human *SCN1A* K1270T mutation (Fig. 3*C*) and was found to cause a semidominant, heat-induced seizure phenotype when exposed to 40°C (Sun et al., 2012).

**Figure 5.**
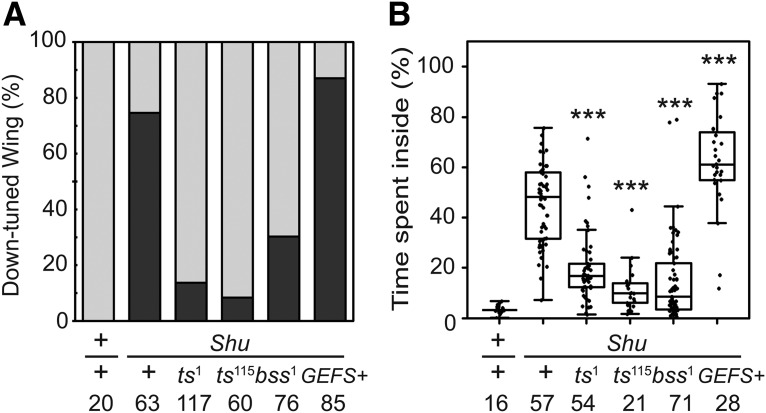
Functional interactions among *Shu* and other mutant alleles of *para.* ***A***, Effects of different *para* mutations on *Shu* morphological phenotype. Indicated are percentages of wild-type (+/+), *Shu* (*Shu*/+), and trans-heterozygous (*Shu*/*para^mutant^*) females exhibiting the normal wing posture (gray) and the downturned wing phenotype (black). ***B***, Effects of different *para* mutations on *Shu* behavioral phenotype. Percentages of time spent in the center of the chamber are shown for wild-type (+/+), *Shu* (*Shu*/+), and trans-heterozygous (*Shu*/*para^mutant^*) females. The Mann–Whitney *U* test was used to assess the effect of each *para* allele on the behavioral phenotype of *Shu*. The statistical significance and sample numbers (*n*) are indicated. ***p < 0.001.

*Shu* mutant females were crossed to *para^ts1^*, *para^ts115^*, *para^bss1^*, or *para ^GEFS+^* males, and the female progeny trans-heterozygous for *Shu* and another *para* mutant allele (i.e., *Shu*/*para^mutant^*) were subjected to phenotypic analysis. We found that the defect in wing posture was significantly suppressed in *Shu* females trans-heterozygous for either *para^ts1^* or *para^ts115^* (Fig. 5*A*). Consistently, *Shu*/*para^ts1^* and *Shu*/*para^ts115^* flies showed improved behavioral phenotypes, spending considerably less time in the center of the chamber than *Shu* females (*p* < 0.001; Fig. 5*B*). Because a previous report demonstrated that *para^ts1^* and *para^ts115^* show hypomorphic function even at permissive temperature (Ganetzky, 1984), these results indicated that a general suppression of *para* function reduces the severity of the morphological and behavioral phenotypes of *Shu* mutants. On the other hand, the phenotypes of *Shu* mutants were not suppressed when *Shu* was combined with *para^GEFS+^* (Fig. 5*A*, *B*). Rather, *Shu*/*para^GEFS+^* flies displayed a more severe behavioral phenotype than *Shu* alone (*p* < 0.001, Fig. 5*B*). A previous study had reported that the knock-in GEFS+ mutation *para^GEFS+^* studied here reduces the activation threshold regardless of temperature (Sun et al., 2012). The worsening effect of *para^GEFS+^* on *Shu* phenotypes is thus consistent with the hypomorphic alleles, *para^ts1^* and *para^ts115^*, having a suppressive effect. In this context, it was unexpected that *Shu*/*para^bss1^* displayed significantly suppressed morphological (Fig. 5*A*) and behavioral (*p* < 0.001; Fig. 5*B*) phenotypes relative to *Shu*/+ flies, because, unlike *para^ts1^* or *para^ts115^*, *para^bss1^* was reported to be a gain-of-function allele (Parker et al., 2011).

### Neuron-specific knockdown of *para* leads to suppression of the *Shu* phenotypes

**Figure 6.**
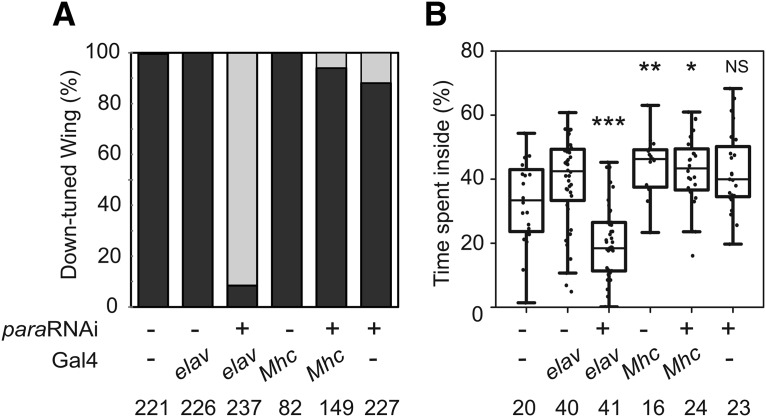
Neuron-specific expression of *para* RNAi significantly improves the *Shu* mutant phenotypes. ***A***, Effects of neuron-specific knockdown of *para* expression on *Shu* morphological phenotypes. Indicated are percentages of control and *para* RNAi-expressing flies displaying the normal wing posture (gray) and the downturned wing phenotype (black). ***B***, Effects of neuron-specific knockdown of *para* expression on *Shu* behavioral phenotypes. Percentages of time spent in the center of the chamber are shown for control and *para* RNAi-expressing flies. The Mann–Whitney *U* test was used to assess the effect of each *para* allele on the behavioral phenotype of *Shu*. *elav*, neuron-specific *elav*-Gal4; *Mhc*, muscle-specific *Mhc*-Gal4. The statistical significance and sample numbers (*n*) are indicated. *p < 0.05; **p < 0.01; ***p < 0.001.

The observation that *para* hypomorphic alleles partially rescued the phenotypes of *Shu* mutants (Fig. 5) prompted us to examine the effect of reduced *para* expression on the *Shu* phenotypes. *Shu* mutants were subjected to knockdown of *para* expression using UAS-*para-RNAi* lines obtained from Vienna Drosophila Resource Center (GD6131 and GD6132). Flies expressing GD6131 *para-RNAi* in neurons exhibited slightly delayed eclosion, but the adults appeared healthy. Interestingly, this genetic manipulation in *Shu* mutants resulted in nearly complete rescue of the downturned wing phenotype (Fig. 6*A*). The behavioral phenotype was drastically improved as well (Fig. 6*B*). Expression of *para-RNAi* in muscle cells using the muscle-specific driver *Mhc*-Gal4 did not lead to improvement of any aspects of the mutant defects examined. In support of the results with GD6131, we observed similar rescue effects when using another *para*-*RNAi* line, GD6132, to suppress *para* expression in *Shu* mutants (data not shown).

### Lithium reduces the severity of *Shu*’s behavioral phenotypes

**Figure 7.**
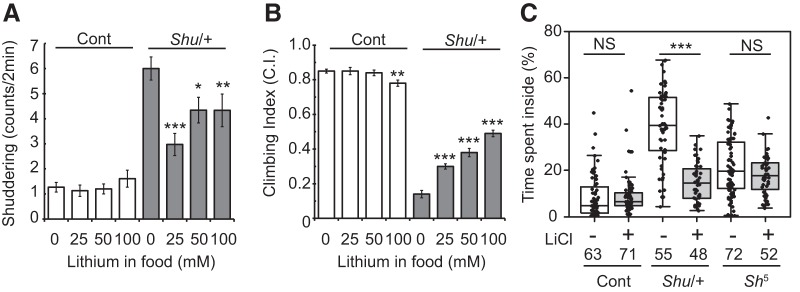
Lithium reduces the severity of behavioral phenotypes of *Shu* mutants. ***A***, Shuddering behavior in wild-type (open) and *Shu* females (closed) after 5-d treatment with food containing 0, 25, 50, or 100 mm LiCl. Data are mean ± SEM. *n* ≥ 30. The statistical significance of differences between the control and each lithium treatment was assessed using the Mann–Whitney *U* test. *p < 0.05; **p < 0.01; ***p < 0.001. ***B***, Reactive climbing ability of wild-type (open) and *Shu* females (closed) after the same LiCl treatment. Data are mean ± SEM of 10 groups of 20 flies. The statistical significance of differences between the control and each lithium treatment was assessed using the Student’s *t*-test. ***p < 0.001. ***C***, Percentages of time spent in the center of the chamber by wild-type (Cont), *Shu* (*Shu*/+), and *Sh^5^* females with or without lithium treatment. Kruskal–Wallis one-way ANOVA on ranks was performed followed by post hoc analysis using Dunn’s method. Sample numbers (*n*) and the statistical significance between lithium-treated and control flies are indicated. ***p < 0.001; NS, not significant (*p* > 0.05).

One of the remarkable observations by Williamson (1982) was that the behavioral phenotypes of *Shu/FM6* flies were significantly suppressed by feeding the mutants lithium-containing food. To examine the effect of lithium on outcrossed *Shu* mutants, we fed the adult flies food supplemented with different concentrations of LiCl. Consistent with the previous report (Williamson, 1982), a 5-d treatment with 100 mm LiCl during adulthood reduced the frequency of shuddering (Fig. 7*A*). In addition, food containing lower concentrations of LiCl (25 and 50 mm) was also effective in suppressing shuddering behavior (Fig. 7*A*). The reduction in shuddering in response to lithium is not due to a general suppression of motor activity, because the same treatment considerably increased coordinated motor activity of *Shu* mutants in a reactive climbing assay (Fig. 7*B*). This improvement was observed in a dose-dependent manner, with higher climbing indices correlating with an increase in LiCl concentration (25, 50 and 100 mm) in the food. In sharp contrast to the normalizing effects of lithium on *Shu* mutant behavior, wild-type flies remained largely unaffected. However, after 5 d of receiving the highest concentration of lithium tested, wild-type flies began to display subtle signs of retarded locomotion (Fig. 7*B*).

Consistently, *Shu* mutants fed food supplemented with 100 mm lithium spent significantly less time in the center of the chamber compared with those maintained on standard food (*p* < 0.001; Fig. 7*C*). The potassium channel mutant, *Sh^5^*, also exhibited jerking and twitching, albeit less frequently and less intensely than *Shu*. Like *Shu*, *Sh^5^* mutants spent more time in the center than wild-type flies (control vs. *Shu^5^*, no lithium, *p* < 0.001). However, unlike *Shu*, these same parameters in *Sh^5^* were not significantly altered by lithium treatment (Fig. 7*C*).

As shown in Figure 1*F*, *Shu* mutants were short-lived. Although lithium had a rescue effect on *Shu* behavioral phenotypes (Fig. 7), it did not positively impact their longevity. Rather, *Shu* lifespan was decreased when adults were maintained on food containing 50 and 100 mm LiCl. Furthermore, even at the lower concentrations of lithium supplementation (i.e., 1 and 10 mm), the drug failed to extend the lifespan of the mutants. The median survival times of *Shu* mutants in this set of experiments were 13 (*n* = 100), 22 (*n* = 150), 36 (*n* = 172), 30 (*n* = 147), and 44 (*n* = 139) d for 100, 50, 10, 1, and 0 mm LiCl.

### *Shu* mutants accumulate higher levels of lithium

Although lithium treatment partially rescued behavioral phenotypes of *Shu* mutants, it had little or even a worsening effect on the behaviors of wild-type flies (Fig. 7*A–C*). To determine whether there was any difference between the two genotypes with respect to physiological responses to lithium, the internal concentrations of lithium in flies fed lithium-containing food for 5 d were analyzed. *Shu* mutants were found to accumulate higher levels of lithium than wild-type flies when fed food containing either 25 or 50 mm LiCl (*p* < 0.05; Table 3). A similar trend was observed when 100 mm LiCl was used, although the difference between the genotypes was not statistically significant. Our wild-type data were comparable to internal lithium concentrations determined in previous studies under similar conditions (Padiath et al., 2004; Dokucu et al., 2005).

**Table 3.** Internal lithium levels in control and *Shu* mutants after treatment with different concentrations of LiCl.

Fly	25 mm LiCl	50 mm LiCl	100 mm LiCl
Control	0.176 ± 0.011	0.303 ± 0.053	0.614 ± 0.11
*Shu*/+	0.321 ± 0.044*	0.503 ± 0.055*	0.935 ± 0.15

Lithium levels in *CS* and *Shu* females after 5 d of treatment with 25, 50, and 100 mm LiCl. Internal lithium levels were elevated in LiCl-treated (25 or 50 mm) *Shu* flies relative to wild-type counterparts. Data are mean ± SEM of three independent experimental groups of 20 flies. *p < 0.05; Student’s *t*-test.

### Different *para* mutants are affected by lithium in an allele-specific manner

**Figure 8.**
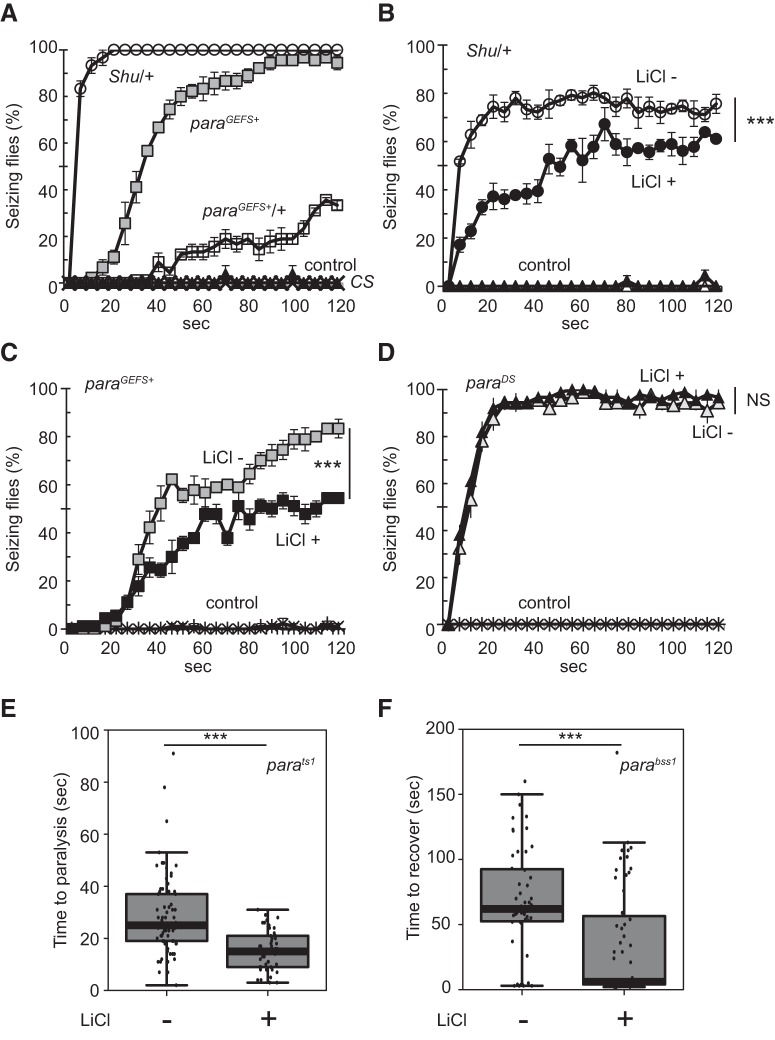
*Shu* mutants and GEFS^+^ flies exhibit a heat-induced seizure phenotype that is suppressed by lithium administration. ***A***, Individual flies were put into glass vials that were submerged in a water bath (40°C) for 2 min. Each fly was examined for seizure status (seizing or not seizing) every 5 s. Three independent experiments were carried out, and 10–30 flies were analyzed in each experiment. The average percentages of seizing flies at each time point (±SEM) are shown for *Shu* (*Shu*/+) and *para^GEFS+^* mutants (homozygotes and heterozygotes). *Shu* mutants showed a heat-induced seizure phenotype similar to, but much more robust than, that of *para^GEFS+^* mutants. The average percentages of seizing flies at each time point (± SEM) are shown for *Shu* mutants at 37°C (***B***), *para^GEFS+^* mutants (homozygotes) at 40°C (***C***), and *para^DS^* mutants (homozygotes) at 38°C (***D***) after treatment with food with (+) or without (–) 100 mm LiCl. Three independent experiments were carried out, and 27–30 flies were analyzed in each experiment. Control flies (*CS* or the genetic background strain for *para^GEFS+^* and *para^DS^* flies) did not seize under these conditions. LiCl treatment significantly suppressed the heat-induced seizure phenotype of *Shu* and GEFS^+^ mutants (two-way repeated-measures ANOVA, Holm–Sidak multiple comparisons, ***p < 0.001). ***E***, Time required for control (*n* = 73) and 100 mm lithium–treated (*n* = 46) *para^ts1^* mutants to become paralyzed at 34°C. LiCl treatment significantly enhanced the heat-induced paralysis phenotype. ***F***, Time required for control (*n* = 51) and 100 mm lithium–treated (*n* = 56) *para^bss1^* mutants to recover from mechanical shock-induced paralysis. LiCl-treated *para^bss1^* mutants displayed reduced recovery time. Mann–Whitney *U* test. ***p < 0.001.

As mentioned above, the *para* mutation in a *Drosophila* GEFS+ model (*para^GEFS+^*) is located near the site of the *Shu* mutation (Fig. 3*C*; K1330T vs. M1327I), presenting the possibility that *Shu* might also exhibit heat-induced seizures. Therefore, we tested *Shu* mutants and found that they respond to increased temperature in a manner similar to that previously observed in GEFS+ flies—albeit in a much more severe manner. Seizure-like behaviors were induced in almost all *Shu* mutants within 30 s of exposure to 40°C, whereas during the same time frame, only a few *para^GEFS+^* heterozygotes and <50% of *para^GEFS+^* homozygotes showed the phenotype (Fig. 8*A*). Unlike GEFS+ flies, *Shu* mutants exhibited seizures even at 37°C (Fig. 8*B*). As was the case for other behavioral abnormalities of *Shu* mutants, the heat-induced phenotype was alleviated when the flies were fed food containing 100 mm lithium (Fig. 8*B*). Furthermore, lithium had a comparable rescue effect on *para^GEFS+^* homozygotes, significantly reducing the severity of their heat-induced seizure phenotype (Fig. 8*C*).

Because lithium feeding suppressed the phenotype of *para^GEFS+^*, we wondered whether phenotypic severity of other *para* mutants was similarly improved by lithium. *para^DS^* is a fly model of human epilepsy generated by Schutte et al. (2014). This knock-in allele carries the mutation in *para* (S1291R) that corresponds to the human *SCN1A* mutation (S1231R) causing a severe seizure disorder termed Dravet syndrome (DS). *Drosophila para^DS^* mutants exhibit heat-induced seizures with onset temperature lower than *para^GEFS+^* mutants (Schutte et al., 2014). We examined the heat-induced phenotype of *para^DS^* with or without feeding 100 mm lithium and found that lithium had no rescue effect (Fig. 8*D*). Likewise, lithium treatment did not lead to phenotypic improvement in *para^ts1^* mutants. Rather, *para^ts1^* mutants paralyzed in shorter time at high temperature when they were treated with lithium (Fig. 8*E*). In contrast, *para^bss1^* mutants were found to recover from mechanical shock–induced paralysis more rapidly when they were fed with lithium-containing food (Fig. 8*F*). In summary, among *para* mutant alleles examined in this study, lithium improved the phenotypes of *Shu*, *para^GEFS+^*, and *para^bss1^* but not those of *para^DS^* and *para^ts1^*.

### Effects of lithium on abnormal seizure-like discharge phenotypes of *Shu* mutants

**Figure 9.**
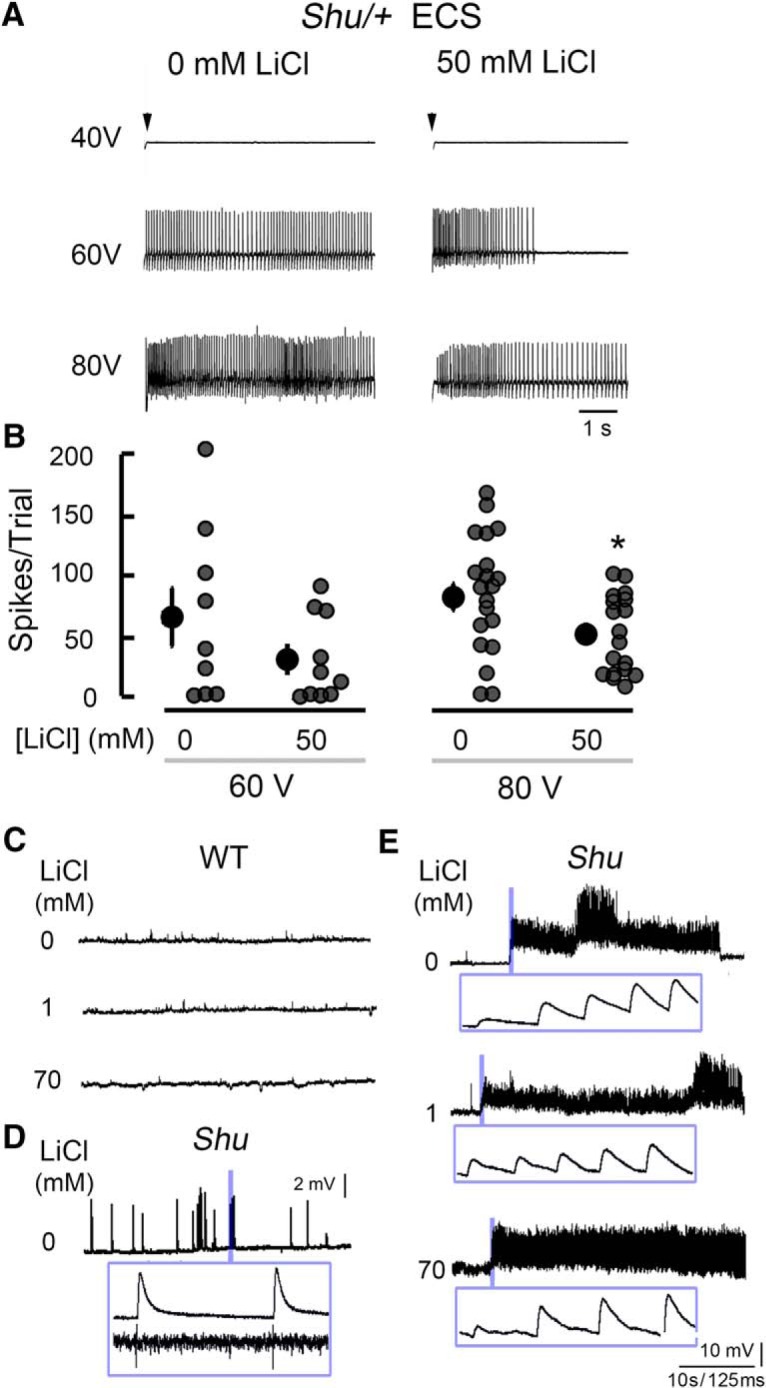
Chronic and acute effects of lithium on the severity of electrophysiological phenotypes of *Shu* mutant adults and larvae. ***A***, Sample traces of ECS discharges (stimulation voltage as indicated) in control *Shu*/*+* flies (0 mm) and lithium-treated *Shu*/*+* flies (50 mm). ***B***, Feeding *Shu*/*+* flies LiCl-supplemented food reduced ECS-triggered spikes per discharge in *Shu*/*+* flies. At 60-V stimulation, the trend was not significant (*p* = 0.24), whereas at 80 V, the ECS discharges were significantly reduced in lithium-treated *Shu*/*+* flies (*p < 0.05, Kruskal–Wallis one-way ANOVA, rank-sum post ho*c* test). ***C***, Acute extracellular LiCl application in wild-type larvae did not evoke spontaneous EJP activities (<10 min). 1 and 70 mm LiCl was added, with NaCl levels adjusted to maintain ionic strength. All larval recordings were done with extracellular Ca^2+^ at 0.5 mm. ***D***, *Shu* homozygous larvae displayed spontaneous discharges of EJPs, associated with motor axon firing (5–20 Hz). ***Inset***, Individual EJPs were coupled with a motor axon action potential. ***E***, Representative traces of *Shu* neuromuscular activity upon extracellular application of Li^+^ at 1 and 70 mm. The spontaneous firing phenotype did not weaken upon application of LiCl. ***Inset***, Expanded EJP traces at the onset of spontaneous activities.

The apparent behavioral improvement after lithium treatment prompted us to examine DLM activity phenotypes of *Shu* mutants in this context to determine how each was affected. We compared flies fed 50 mm LiCl to those receiving control food and found that LiCl feeding influenced only selected electrophysiological phenotypes. For instance, it did not significantly reverse the characteristic spontaneous DLM activity seen in *Shu* flies (mean spike rates for control vs. LiCl fed: 0.97 Hz, *n* = 8, vs. 0.93 Hz, *n* = 10, *p* = 0.36, rank sum test). However, for ECS-triggered discharges, the number of DLM spikes per discharge was reduced in LiCl**-**fed *Shu* flies compared with controls (Fig. 9*A*). At a stimulus intensity of 80 V, LiCl**-**fed *Shu* flies displayed discharges that were significantly milder in terms of spike count compared with control *Shu* flies (Fig. 9*B*; *p* < 0.05). A tendency of reduced spike numbers was observed at 60**-**V stimulation, although the difference did not reach statistical significance. Therefore, our electrophysiological observations were consistent with the behavioral evidence that LiCl feeding can improve aspects of the seizure phenotypes of *Shu* flies.

Behavioral suppression of adult *Shu* mutants required feeding of lithium for a few days to observe chronic improvements (Williamson, 1982). Similarly, DLM physiology also indicated a lack of acute effects immediately after lithium administration. To directly assess the acute effects of lithium application, we used the larval neuromuscular preparation and observed action potential and neuromuscular transmission. In contrast to wild-type larvae, which did not display spontaneous EJPs with motor axons severed from the CNS (Fig. 9*C*), the surviving third-instar *Shu*/*Shu* larvae displayed extreme hyperexcitability, as indicated by spontaneous discharges of EJPs, correlated with bursting of motor axon action potentials, known to be mediated by *para* Na_v_ channels (Fig. 9*D*). Addition of 1 mm LiCl to the saline did not cause acute suppression in excitability (abnormal spontaneous EJPs persisted up to 20 min; Fig. 9*E*). This concentration of lithium is approximately comparable to the internal concentration after LiCl feeding to adult flies with 100 mm in the medium (Padiath et al., 2004; Dokucu et al., 2005). Effective serum lithium concentrations for treating patients with BPD are also in a range of ∼1 mm (Severus et al., 2008). In classic studies, Li^+^ is known to block the Na^+^/K^+^ ATPase pump but can also serve as a highly efficient charge carrier through Na_v_ channels, with permeability higher than Na^+^ (Hille, 2001). We replaced Na^+^ with Li^+^ in saline (70 mm) and found that this drastic treatment did not cause any substantial acute effect on hyperexcitability. Further, even after prolonged incubation (up to 20 min), it only led to a new pattern of extreme hyperexcitability, i.e., giant plateaued EJPs (not shown), reminiscent of those in *eag Sh* hyperexcitable K^+^ channel double mutants caused by high-frequency bursting of motor axon action potentials (Ganetzky and Wu, 1982). Consistently, LiCl at 1 or 70 mm did not produce any acute effect on wild-type larvae to generate spontaneous EJPs (Fig. 9*C*).

### Effects of lithium treatment on gene expression in the adult heads

Our findings suggested that lithium’s suppressive effect on *Shu* phenotypes is not due to acute actions of lithium but rather through long-term physiological adjustments to lithium treatment. Such adjustments possibly involve alterations in gene expression. To explore this possibility, we performed microarray analysis and investigated the effect of the *Shu* mutation and lithium treatment on genome-wide gene expression in adult heads. Gene expression profiles were compared between genotypes (wild-type and *Shu*) and treatment (with or without lithium treatment) using Affymetrix GeneChip *Drosophila* Genome 2.0 Arrays as described in our previous study (Kasuya et al., 2009b). Three biological replicates were tested for each condition and showed high correlation coefficients (*R* > 0.93), indicating that the experimental data were sufficiently reproducible and reliable.

**Figure 10.**
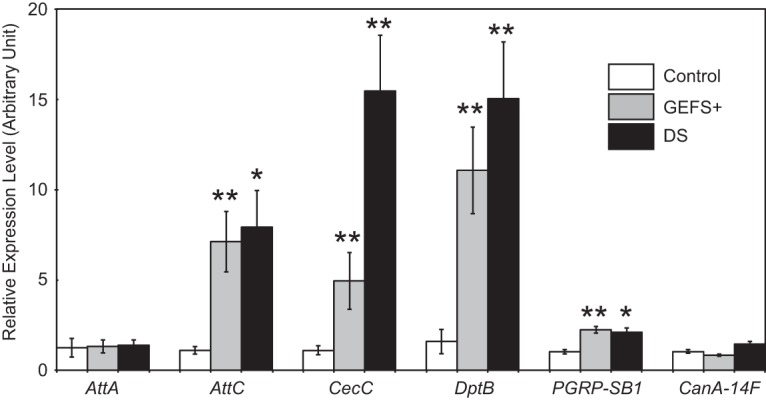
Expression of innate immune response genes is upregulated in *para^GEFS+^* and *para^DS^* mutants. Expression levels of the selected innate immune response genes were examined in control flies and two *para* knock-in mutants, *para^GEFS+^* and *para^DS^*, by quantitative real-time RT-PCR. Data are normalized transcript levels presented as mean ± SEM of five to eight independent experiments. *p < 0.05; **p < 0.001.

When gene expression profiles were compared between *Shu* mutants and genetically matched wild-type flies, 17 genes displayed a significant difference (*p* < 0.05, Welch *t*-test) in transcript levels with a fold change >2 (Table 4). Among them, 14 and three genes were up- and downregulated in *Shu* mutants, respectively. Intriguingly, seven of the 14 upregulated genes are directly involved in the innate immune response (Table 4). These include six genes encoding for antimicrobial peptides (AMPs; Lemaitre and Hoffmann, 2007): *Diptericin B* (*DptB*), *Attacin A* (*AttA*), *Attacin B* (*AttB*), *Attacin C* (*AttC*), *Cecropin B* (*CecB*), and *Cecropin C* (*CecC*). *PGRP-SB1*, another upregulated gene in *Shu*, encodes for a peptidoglycan recognition protein that functions upstream of the signaling cascades regulating the systemic production of AMPs (Lemaitre and Hoffmann, 2007). In addition to these bona fide immune genes, *CG42807 and CG32368* were significantly upregulated in *Shu*. Although the function of these genes is not known, they were reported as two of the most upregulated genes in response to the endogenous presence of microbiota. They are tentatively annotated to encode small peptides, potentially serving as novel immune effectors (Broderick et al., 2014). Furthermore, one of the three calcineurin isoforms, *calcineurin A at 14F* (*CanA-14F*) was upregulated in *Shu*. *CanA-14F*, along with the other two calcineurin genes in *Drosophila*, has been shown to play a role in innate immune signaling (Dijkers and O’Farrell, 2007; Li and Dijkers, 2015). Upregulation of immune gene expression was also observed in two seizure-prone *para* knock-in mutants, *para^DS^* and *para^GEFS+^*. They displayed elevated expression of *AttC*, *CecC*, *DptB*, and *PGRP-SB1*. However, unlike *Shu*, these mutants did not show higher expression of *AttA* and *CanA-14F* compared with genetically matched control flies (Fig. 10).

**Table 4.** List of genes whose expression level is significantly altered by the *Shu* mutation.^a^

Gene symbol	Cytogenetic location	Fold change	*p*-value^b^	Function^c^
*DptB*	55F8	↑12.2	0.0343	Defense response (antibacterial peptide)
*AttC*	50A3	↑8.94	0.0471	Defense response (antibacterial peptide)
*CecC*	99E2	↑8.22	0.0423	Defense response (antibacterial peptide)
*AttB*	51C1	↑7.99	0.0170	Defense response (antibacterial peptide)
*CG31809*	36B2	↑6.57	0.0009	Steroid dehydrogenase activity
*AttA*	51C1	↑6.31	0.0172	Defense response (antibacterial peptide)
*Ste12DOR*	12D2	↑3.33	0.0342	Protein kinase CK2 activity
*CecB*	99E2	↑3.17	0.0159	Defense response (antibacterial peptide)
*PGRP-SB1*	73C1	↑2.78	0.0300	Defense response (peptidoglycan binding)
*CG42807*	50B2	↑2.31	0.0246	Unknown
*CanA-14F*	14F	↑2.21	0.0109	Protein serine/threonine phosphatase activity
*CG32368*	66A19	↑2.20	0.0028	Unknown
*Ccp84Aa*	84A	↑2.13	0.0008	Structural constituent of chitin-based cuticle
*CG31272*	86C5	↑2.04	0.0283	Transporter/lipase activity
*CG9377*	34B7	↓14.3	0.0488	Serine-type endopeptidase activity
*CG31116*	86F8	↓3.57	0.0163	Voltage-gated chloride channel activity
*Nox*	53B3	↓2.28	0.0176	Electron carrier activity; oxidoreductase activity

^a^Genes that were detected on all three chips for either wild-type (Kasuya et al., 2009b) or *Shu* RNA samples and differentially regulated in *Shu* with a fold change *>*2 and *P* < 0.05. Gene ranking is based on amount of fold change. ^b^Determined by Welch’s *t-*test (see Materials and Methods). ^c^Based on FlyBase.

**Figure 11.**
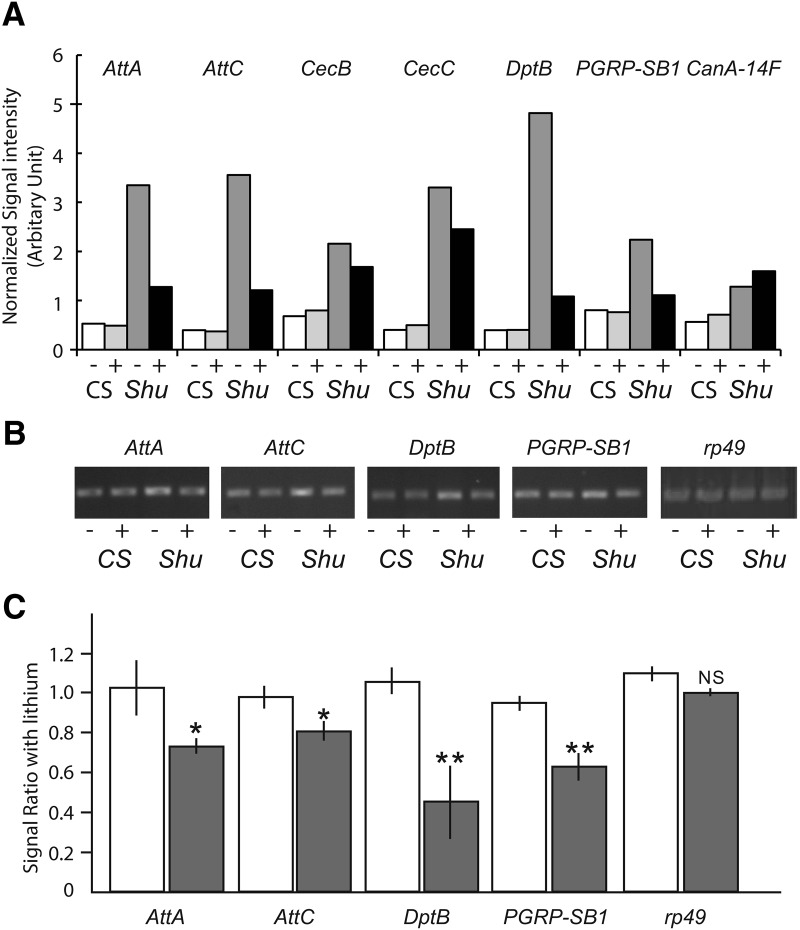
Lithium suppresses the increased expression of innate immune response genes in *Shu* mutants. ***A***, Expression levels of innate immune response genes and *CanA-14F* in wild-type females from microarray analysis were compared with those in *Shu* mutants with or without 50 mm lithium treatment for 24 h. Data are presented as normalized average signal intensities of three biological replicates for each condition. Expression levels of the selected innate immune response genes were examined by RT-PCR in wild-type and *Shu* mutants with or without 50 mm lithium treatment for 24 h. A representative agarose gel (***B***) and ratios between transcript levels in control and lithium-treated flies based on pixel intensity of the bands (***C***) are shown (*CS*, open bar; *Shu*/+, closed bar). Data are mean ± SEM of three independent experiments. *p < 0.05; **p < 0.001; NS, not significant.

We next examined whether lithium treatment has any effect on elevated levels of immune gene expression in *Shu*. As shown in Figure 11*A* and *B*, treatment with lithium had a general propensity to normalize expression of immune-related genes, although *CanA-14F* transcript levels in *Shu* remained higher than those in wild-type flies even after lithium treatment. Quantitative analysis of RT-PCR results confirmed that AMP genes and *PGRP-SB1* were down-regulated by lithium treatment in *Shu* mutants but not in the wild-type flies (Fig. 11*C*).

We analyzed microarray data to further assess the effect of lithium on genome-wide gene expression in *Shu*. When a comparison was made between *Shu* mutants with and without lithium treatment, 20 and 5 genes were up- and down-regulated by lithium with *p* < 0.05 and a fold change >2 (Table 5). Using similar criteria, we previously reported that 11 genes were most significantly up-regulated by lithium treatment in wild-type flies (Kasuya et al., 2009b). Noticeably, 9 of the 11 upregulated genes in wild-type flies were also found to be upregulated in *Shu*. These include genes potentially involved in amino acid transport and metabolism (*Lithium-inducible SLC6 transporter*/*List* and *CG1673*), detoxification and stress response (*CG5999* and *Activity-regulated cytoskeleton associated protein 1*/*Arc1*), folate-dependent metabolism and ROS regulation (*NAD-dependent methylenetetrahydrofolate dehydrogenase*/*Nmdmc*), and oxidation-reduction process (*Cyp309a1*; Flybase). Although lithium-inducible genes are largely shared by *Shu* mutants and wild-type flies, none of the significantly downregulated genes were found to be in common (Kasuya et al., 2009b). The five genes downregulated by lithium in *Shu* include two antibacterial or antifungal peptide genes (*Diptericin B*/*DptB* and *Metchnikowin*/*Mtk*) and two oxidoreductase genes (*deadhead*/*dhd* and *CG5653*; Flybase).

**Table 5.** List of genes whose expression level is significantly altered by lithium treatment in *Shu* mutants.^a^

Gene symbol	Cytogenetic location	Fold change	*p*-value^b^	Function^c^
*List*	55E10	↑7.11	0.0001	Sodium:neurotransmitter symporter
*CG5999*	87C8	↑6.55	0.0462	UDP-glucosyltransferase activity
*Arc1*	50F6	↑4.67	0.0002	Stress response
*CG7763*	47F11	↑3.25	5.3 × 10^−6^	C-type lectin
*MtnD*	92F1	↑3.14	0.0445	Metal homeostasis
*Nmdmc*	85C3	↑2.92	0.0033	NAD-dependent methylenetetrahydrofolate dehydrogenase activity
*CG1673*	11F1	↑2.90	0.0026	Branched-chain amino-acid aminotransferase activity
*Bin1*	89B7	↑2.74	0.0126	Histone deacetylase complex
*Hsp22*	67B2	↑2.59	0.0003	Response to oxidative stress
*Lsp1γ*	61A6	↑2.42	0.0094	Nutrient reservoir activity
*Cyp309a1*	22F4-23A1	↑2.29	0.0034	Cytochrome P450; oxidation-reduction process
*CG11425*	79E4	↑2.28	0.0055	hydrolase
*CG15784*	4F10	↑2.26	0.0014	Unknown
*Blos2*	36C11	↑2.19	0.0415	Biogenesis of lysosome-related organelles
*NimC2*	34E5	↑2.17	0.0162	Phagocytosis
*Cpr64Ac*	64A10	↑2.15	0.0023	Cuticular protein
*Ahcy13*	13C3	↑2.14	0.0001	Adenosylhomocysteinase activity
*CG34136*	39A1	↑2.07	0.0137	Unknown
*AOX1*	88F7	↑2.06	0.0042	Aldehyde oxidase activity
*Ama*	84A5	↑2.04	0.0126	Axon pathfinding
*dhd*	4F4	↓6.33	0.0156	Thioredoxin
*DptB*	55F8	↓4.44	0.0487	Defense response (antibacterial peptide)
*Mtk*	52A1	↓2.85	0.0371	Defense response (antimicrobial peptide)
*CG7227*	28D3	↓2.46	0.0235	Defense response (scavenger receptor activity)
*CG5653*	66E5	↓2.18	0.0083	Amine oxidase activity

^a^Genes that were detected on all three chips for RNA samples of *Shu* with or without lithium treatment and differentially regulated under these conditions with a fold change *>*2 and *p* < 0.05. Gene ranking is based on amount of fold change. ^b^Determined by Welch’s *t-*test (see Materials and Methods). ^c^Based on FlyBase.

We also applied a DAVID functional annotation tool (https://david.ncifcrf.gov/tools.jsp) to identify biological pathways likely associated with changes in lithium-induced gene expression. Using less stringent criteria (false discovery rate <0.5 and fold change >1.2) than the aforementioned analysis, 158 genes were identified as differentially regulated genes in *Shu* with and without lithium treatment. Analysis using the DAVID functional annotation chart program identified 19 partially redundant gene ontology (GO) term entries as highly enriched in a list of lithium-responsive genes with *p* < 10^−5^ (Table 6). Interestingly, amino acid metabolism represented by multiple GO terms (e.g., 0008652, 0046394, 0006564, 0009066) stood out as the biological process most significantly affected by lithium treatment. Genes involved in carbohydrate metabolism and redox reactions were also found to be strongly affected when *Shu* mutants were treated with lithium.

**Table 6.** Functional annotation chart of selected lithium-responsive genes analyzed by DAVID.

Enriched Gene Ontology (GO) annotation terms	GO ID	*p*-value^a^	Fold enrichment
Cellular amino acid biosynthetic process	0008652	5.1 × 10^−8^	21.7
Carboxylic/organic acid biosynthetic process	0046394/0016053^b^	8.6 × 10^−8^	12.3
Amine biosynthetic process	0009309	1.0 × 10^−7^	15.0
Cofactor binding	0048037	5.5 × 10^−7^	5.4
Oxidation-reduction process	0055114	1.8 × 10^−6^	3.0
Lipid particle	0005811	2.4 × 10^−6^	4.9
L-serine biosynthetic process	0006564	7.7 × 10^−6^	78.6
Hexose metabolic process	0019318	1.0 × 10^−5^	8.3
Aspartate family amino acid metabolic process	0009066	1.6 × 10^−5^	30.2
L-serine metabolic process	0006563	1.9 × 10^−5^	62,9
Monosaccharide metabolic process	0005996	2.7 × 10^−5^	7.3
Mitochondrion	0005739	2.8 × 10^−5^	2.9
Cellular nitrogen compound biosynthetic process	0044271	3.3 × 10^−5^	4.4
Serine family amino acid metabolic process	0009069	3.8 × 10^−5^	24.6
IMP biosynthetic/metabolic process	0006188/0046040^c^	6.5 × 10^−5^	44.9
Serine family amino acid biosynthetic process	0009070	6.5 × 10^−5^	44.9
Transaminase activity	0008483	7.4 × 10^−5^	20.8

^a^*p*-value for a modified Fisher exact test (EASE score). ^b,c^The identical genes are assigned to these terms for the *Drosophila* genome.

**Table 7.** Primers used for PCR experiments.

Gene symbol	Forward primer (5′–3′)	Reverse primer (5′–3′)
Real-time RT-PCR		
*AttA*	TGGCAATCCCAACCACAA	CATTGTTGTAGGCCAAAGTTCC
*AttC*	AACTCCCGATCACCATGTTATT	CAGGCCGTGTCCATGATT
*CecC*	CGGTTGGCTGAAGAAACTTG	GCAATTCCCAGTCCTTGAATG
*DptB*	ACTGGCATATGCTCCCAATTT	TCAGATCGAATCCTTGCTTTGG
*PGRP-SB1*	GATGAACACATCAACGGCAATTA	TGCTGCGTGGTTCAATCT
*rp49*	CCAGTCGGATCGATATGCTAAG	CCGATGTTGGGCATCAGATA
Semiquantitative RT-PCR		
*AttA*	ACGGTCCACTCGTCCACTTG	CAATGCTGGTCATGGTGCCTC
*AttC*	CAGATCGGTCTGGCCCTTGAA	TCATGGACACGGCCTGGAAC
*DptB*	TGTAGCTTCTGAAGTGCCCT	TTCATTGGACTGGCTTGTGC
*PGRP-SB1*	TTGTCTGCCGATGACCGAACA	TCTATCCGCCAATGCCCTGC
*rp49*	TTCGCTAAGCAGTAGCTGCGAC	GTTAACACGCAGGCGACGGAA

## Discussion

In this study, we identified *Shu*, a *Drosophila* mutation causing severe behavioral defects, as a novel hypermorphic allele of the Na_v_ channel gene *para* (Fig. 3). The notion that the *Shu* mutation is hypermorphic is based on *Shu*’s morphological, electrophysiological, and behavioral phenotypes (Figs. 1 and 2) as well as the observation that *Shu* phenotypes were substantially suppressed when *para* function was reduced using *para* hypomorphs (Fig. 5) or *para*-RNAi (Fig. 6). These results suggest that the *Shu* Na_v_ channels cause an increase in sodium currents, and that this defect is compensated by reduced *para* function, leading to a more balanced neuronal output.

We were able to at least partly recapitulate *Shu* phenotypes by driving expression of *Shu* Na_v_ channels in wild-type flies (Fig. 4). However, the effect of the *Shu* transgene was mild when the motor neuron-positive driver C164-Gal4 was used (Fig. 4), and there was little effect with a pan-neuronal driver *elav*-Gal4. Our result somewhat resembles that of a previous attempt to reproduce the *para^bss1^* mutant phenotype with pan-neuronal expression of the *para^bss1^* transgene (Parker et al., 2011), where only a small subset (1.6%) of wild-type flies expressing the *para^bss1^* transgene displayed the bang-sensitive phenotype. Although the reasons underlying the weak effect of these mutant transgenes remain unknown, a lack of alternative splicing may contribute. Previous studies revealed that 27–29 distinct *para* splice variants exist, and that they are functionally diverse (Olson et al., 2008; Lin et al., 2009). In our study and that of Parker et al. (2011), the cDNA of the Na_v_ 1-1 *para* isoform, which is presumably the most common splice variant in adult flies (Olson et al., 2008; Lin et al., 2009), was utilized to construct the *Shu* and *para^bss1^* transgenes. To completely mimic the effects of these dominant *para* mutations, the transgenes may need to be expressed, in specific cell types, as certain splice variants.

Importantly, as originally reported by Williamson (1982), we observed suppression of the seizure-like phenotypes of *Shu* when the mutants were fed lithium-containing food (Fig. 7). In this study, we also demonstrated that GF motor outputs of *Shu* mutants exhibit hyperexcitable, seizure-like discharges and that lithium partially rescues these phenotypes as well (Fig. 9). Our results show that lithium can improve physiological as well as behavioral defects originally caused by aberrant sodium channel function. Consistently, lithium had a rescue effect on the heat-induced seizure phenotype of *para^GEFS+^* and the bang-sensitive phenotype of *para^bss1^* (Fig. 8*C*, *F*). Based on their electrophysiological phenotypes, both *para^GEFS+^* and *para^bss1^* are considered to be *para* gain-of-function alleles (Parker et al., 2011; Sun et al., 2012). In contrast, lithium does not seem to improve the phenotypes of two *para* loss-of-function alleles, *para^DS^* and *para^ts1^* (Fig. 8*D*, *E*). This apparent correlation between types of Na_v_ channel mutations (i.e., gain- and loss-of-function) and the effects of lithium suggest that lithium may preferentially improve defects caused by aberrantly activated Na_v_ channels.

Interestingly, many antiepileptic drugs targeting Na_v_ channels, such as lamotrigine, are effective for BPD (Landmark, 2008). This therapeutic regimen for BPD suggests that its pathophysiology may be influenced by abnormal Na_v_ channel function. Further evidence for the connection between BPD and Na_v_ channels comes from genome-wide association studies, which have implicated *ankyrin-G* (*ANK3*) as a potential risk factor for BPD (Ferreira et al., 2008). *ANK3* encodes an adaptor protein that regulates Na_v_ channel assembly (Poliak and Peles, 2003). Likewise, a gene expression study in the postmortem brains of BPD patients found that the α- and β-subunits of the voltage-gated type I sodium channel genes were upregulated (Smolin et al., 2012). Overall, these data provide support for the notion that the dysregulation of Na_v_ channel function may play a role in the etiology of BPD, and that some of the most effective pharmacological therapies for this disorder may counteract the effect of aberrant Na_v_ channel activity.

Lithium is commonly thought to elicit its mood-stabilizing effect by inhibiting GSK-3β, IMPase, or IPPase (Manji and Lenox, 1998; Phiel and Klein, 2001; Lenox and Wang, 2003; Machado-Vieira et al., 2009). However, our experiments did not yield results that directly connect lithium’s rescue effect with these enzymes. For instance, pharmacological inhibition of GSK-3β using AR-A014418, which was shown to reduce tau-induced pathology by inhibiting GSK-3β (Mudher et al., 2004), did not suppress behavioral phenotypes of *Shu*. The median values of percentage of time spent inside were 42.8 and 47.9% for control (*n* = 37) and AR-A014418-treated (*n* = 46; *p* > 0.05) flies, respectively. Likewise, feeding *Shu* mutants with L-690,330, which is known to be ∼1000-fold more potent than lithium in inhibiting IMPase (Atack et al., 1993), did not lead to phenotypic suppression either. The median values of percentage of time spent inside were 43.4 and 39.4% for control (*n* = 19) and L-690,330-treated (*n* = 23; *p* > 0.05) flies, respectively. These negative data with the enzyme inhibitors suggest that lithium may act independently of GSK-3β and inositol signaling in exerting its rescue effect on *Shu* mutants.

Early voltage-clamp studies demonstrated that the permeability of Na_v_ channels to Li^+^ is nearly identical to their permeability to Na^+^ itself (*P*_Li_/*P*_Na_ = 0.93; Hille, 1972; Campbell, 1976). In bovine adrenal chromaffin cells, Li^+^ was shown to inhibit radioactive Na^+^ influx through veratridine-activated sodium channels, independently of GSK-3β (Yanagita et al., 2007). Such observations raised the possibility that lithium may suppress activity of *Shu* Na_v_ channels to confer a rescue effect. However, direct application of lithium did not cause acute suppression of hyperexcitability of motor neurons displayed by *Shu* larvae (Fig. 9*E*). Moreover, total replacement of Na^+^ with Li^+^ did not alter the hyperexcitability phenotype in the larval motor axon (Fig. 9*E*). Our results are consistent with the previous observation that Li^+^, like ouabain, can enhance neuromuscular excitability in wild-type larvae via inhibition of Na^+^/K^+^ ATPase (Jan and Jan, 1978), in contrast to the suppression effect of chronic lithium feeding to *Shu* mutants. Furthermore, a recent report demonstrates interesting effects of long-term lithium treatment of induced pluripotent stem cells derived from lithium-responsive BPD patients. The differentiated neurons from such patients display hyperexcitability that can be suppressed after 1-week incubation in lithium-containing culture medium. A reduction in neuronal firing is coupled with a decrease in Na^+^ and K^+^ currents, consistent with a long-term physiological readjustment of neuronal excitability mechanisms (Mertens et al., 2015).

Our microarray and RT-PCR analyses revealed that expression of innate immune response genes is enhanced in the head of *Shu* mutants and that lithium tends to normalize their expression levels (Table 4, Fig. 11). The correlation between immune gene expression and severity of *Shu* phenotypes raises the possibility that the innate immune system is involved in manifestation of *Shu* phenotypes and their modulation by lithium. Changes in immune gene expression were also observed in two other seizure-prone *para* knock-in mutants (Fig. 10) and are associated with genetic suppression of seizure-like phenotypes of *Drosophila easily shocked* (*eas*) mutants (Hekmat-Scafe et al., 2005). In addition, experimental and clinical studies have provided accumulating evidence for a strong relationship between an activated innate immune system and the pathophysiology of psychiatric and neurological disorders, including BPD and human epilepsies, as well as in rodent epilepsy models (Granata et al., 2011; Vezzani et al., 2011; Legido and Katsetos, 2014; Elhaik and Zandi, 2015). Recent studies using mice also demonstrated that lithium can attenuate innate immune responses in a GSK-3β–independent manner both *in vitro* and *in vivo* (Wang et al., 2013).

A combination of microarray and bioinformatics analyses revealed additional genes and biological pathways significantly affected by lithium treatment in *Shu*. Particularly notable are those related to amino acid metabolism and oxidation-reduction processes (Tables 5 and 6). Interestingly, alterations of brain amino acid metabolism are implicated in the antiepileptic effect of the ketogenic diet (Yudkoff et al., 2001), whereas redox reactions, particularly those involved in oxidative stress, are suggested to play critical roles in initiation and progression of epilepsy (Shin et al., 2011). Future experiments are required to determine whether these biological pathways as well as the innate immune system have functional significance in lithium-dependent improvement of *Shu* phenotypes caused by abnormal Na_v_ channel activity.

Despite several decades of scientific investigation, the exact etiology and the mechanisms of drug action for many psychiatric disorders, such as BPD, remain largely unknown. Attempts to better understand the pathophysiology of these illnesses using animal models, including *Drosophila*, continue to be a challenge but also prove highly promising (Nestler and Hyman, 2010). Taken together, our results suggest that *Shu* and other *Drosophila* sodium channel mutants have the potential to be useful and experimentally amenable tools for elucidating the actions of lithium in the nervous system, and that they may ultimately contribute to an understanding of lithium-responsive disorders in humans.

## Synthesis

The decision was a result of the Reviewing Editor Vivian Budnik and the peer reviewers coming together and discussing their recommendations until a consensus was reached. A fact-based synthesis statement explaining their decision and outlining what is needed to prepare a revision is listed below. The following reviewers agreed to reveal their identity: Bing Zhang, Mani Ramaswami

The mechanisms of lithium action in the brain are of wide interest, given the widespread use of lithium to treat neuropsychiatric disorders. The present manuscript provides evidence for a potential function during seizures in a gain of function Na+ channel mutation. Although the studies at the neuromuscular junction are puzzling, revealing that the mechanism of Li action cannot be simply explained, both reviewers agreed that the information provided in this study is likely to be of interest.
